# Production of quorum sensing-related metabolites and phytoalexins during Pseudomonas aeruginosa–Brassica napus interaction

**DOI:** 10.1099/mic.0.001212

**Published:** 2022-08-18

**Authors:** Jamie Cook, Joseph P. M. Hui, Janie Zhang, Michaela Kember, Fabrice Berrué, Junzeng Zhang, Zhenyu Cheng

**Affiliations:** 1 Department of Microbiology and Immunology, Dalhousie University, Halifax, Nova Scotia, Canada; 2 Aquatic and Crop Resource Development Research Centre, National Research Council Canada, Halifax, Nova Scotia, Canada

**Keywords:** *Pseudomonas aeruginosa*, canola, quorum sensing, phytoalexin, metabolomics, LC-HRMS

## Abstract

*Pseudomonas aeruginosa* is an opportunistic bacterial pathogen that has been shown to interact with many organisms throughout the domains of life, including plants. How this broad-host-range bacterium interacts with each of its diverse hosts, especially the metabolites that mediate these interactions, is not completely known. In this work, we used a liquid culture root infection system to collect plant and bacterial metabolites on days 1, 3 and 5 post-*P*. *aeruginosa* (strain PA14) infection of the oilseed plant, canola (*Brassica napus*). Using MS-based metabolomics approaches, we identified the overproduction of quorum sensing (QS)-related (both signalling molecules and regulated products) metabolites by *P. aeruginosa* while interacting with canola plants. However, the *P. aeruginosa* infection induced the production of several phytoalexins, which is a part of the hallmark plant defence response to microbes. The QS system of PA14 appears to only mediate part of the canola*–P. aeruginosa* metabolomic interactions, as the use of isogenic mutant strains of each of the three QS signalling branches did not significantly affect the induction of the phytoalexin brassilexin, while induction of spirobrassinin was significantly decreased. Interestingly, a treatment of purified QS molecules in the absence of bacteria was not able to induce any phytoalexin production, suggesting that active bacterial colonization is required for eliciting phytoalexin production. Furthermore, we identified that brassilexin, the only commercially available phytoalexin that was detected in this study, demonstrated a MIC of 400 µg ml^−1^ against *P. aeruginosa* PA14. The production of phytoalexins can be an effective component of canola innate immunity to keep potential infections by the opportunistic pathogen *P. aeruginosa* at bay.

## Introduction

*Pseudomonas aeruginosa* is a Gram-negative, rod-shaped bacterium that belongs to the bacterial family *Pseudomonadaceae*. It is an opportunistic pathogen with a broad host range, including *Arabidopsis*, *Caenorhabditis elegans*, *Drosophila melanogaster*, mice and humans [1,4]. The pathogenicity of *P. aeruginosa* in humans has been well characterized [5,9]. Since *P. aeruginosa* is found ubiquitously within the environment, this bacterium has also been shown to infect plants. The earliest described *P. aeruginosa* infection occurred in tobacco plants in the Philippines in 1930 [10]. It was found that in tobacco plants, *P. aeruginosa* causes severe leaf spotting and necrosis, as well as soft stem rot in seedlings [10]. Additionally, *P. aeruginosa* was shown to be introduced into hospitals by contaminated vegetables [11]. More recently, the model plant *Arabidopsis thaliana* has been used to characterize host immunity and bacterial pathogenesis of *P. aeruginosa* in plants [1,12,17]. However, little is known about *P. aeruginosa* virulence mechanisms in plant infections.

Quorum sensing (QS) is a bacterial cell-to-cell communication approach using diffusible signalling molecules. QS systems have been shown to play critical roles in the regulation of bacterial virulence [18,19]; however, its contribution to *P. aeruginosa* pathogenicity in plants remains elusive. There are three main QS branches in *P. aeruginosa*: two acyl-homoserine lactone (AHL) systems, which are LasRI and RhlRI, and a quinolone signalling system [20]. The LasRI system produces and responds to *N*-(3-oxododecanoyl) homoserine lactone (3-oxo-C12-HSL), which is produced by the LasI synthase and recognized by the transcriptional regulator LasR [21,22]. The RhlRI AHL system produces and responds to *N*-butanoyl homoserine lactone (C4-HSL), which is produced by the RhlI synthase and sensed by the transcriptional regulator RhlR [23]. The quinolone signalling system is governed by the transcriptional regulator, MvfR (also known as PqsR) and the quinolone signalling molecules, including 4-hydroxy-2-heptylquinoline (HHQ) and 2-heptyl-3-hydroxy-4-quinolone (PQS) [24,26]. HHQ is synthesized in a two-step pathway from fatty acids by the enzymes encoded by the *pqs* biosynthesis genes (*pqsABCD*) [27]. The enzyme PqsH finally converts HHQ into PQS [25]. The QS signalling network in *P. aeruginosa* is particularly complex and coordinately regulates the expression of hundreds of genes, representing over 10 % of its genome, with many overlapping targeted genes among the regulons of each individual QS branch [28,34]. It should be noted that the production of the regulatory elements (both transcription regulators and signalling molecules) of the RhlRI and MvfR–PQS branches were proposed to be hierarchically regulated by the LasRI system [28,29, 35], where LasR positively regulates the transcription of *rhlR*, *rhlI*, *mvfR* and *pqsH* genes. In addition, the RhlRI branch also negatively regulates the MvfR–PQS branch by inhibiting the transcription of the *mvfR* gene and *pqsABCD* operon [36]. A more recent model suggested a circular mode of the *P. aeruginosa* QS signalling systems, where MvfR binds to the promoter regions and directly regulates the expression of *lasR* and *rhlR* genes [32].

QS systems in *P. aeruginosa* control the production and secretion of a large pool of molecules. The following are a few examples of the *P. aeruginosa*-secreted molecules whose expression are under direct regulation of various QS systems. The LasRI system directly regulates the production of many virulence factors, which include the LasA and LasB elastases and exotoxin A [21,37, 38]. The RhlRI system directly regulates the production of rhamnolipids [39]. The MvfR–PQS complex activates the expression of *phzA1-phzG1* operons consisting of genes that are involved in the production of pyocyanin [30,40]. Many of the QS-regulated targets contribute to pathogenicity during *P. aeruginosa* infections. A previous study using a mung bean seedling infection model with *P. aeruginosa* strain PAO1 showed that a QS mutant was significantly less virulent when compared with the WT PAO1 [41]. Moreover, our recent study showed that within the closely related plant hosts *Arabidopsis thaliana* and *Brassica napus* (oilseed plant, canola), mutations in QS caused a higher degree of reduction in *P. aeruginosa* growth in a leaf infiltration model than in a root infection model [42]. However, due to the lack of research of plant*–P. aeruginosa* interactions and the difficulty of metabolomic profiling in the rhizosphere (the soil that is in close proximity to plant roots), there have been no reports on the involvement of QS-related metabolites in *P. aeruginosa* virulence in plants.

On the other side, plant innate immunity plays instrumental roles in defence against pathogens [43]. Upon pathogenic challenge, plants activate various immune signalling pathways that start a cascade leading to the production of a broad array of defence products [43]. Phytoalexins are a group of low-molecular-mass antimicrobial secondary metabolites with diverse structures [44]. There is numerous evidence in support for the role of phytoalexins in plant defence against fungal or bacterial infections [44,45]. The mode of antibacterial mechanism of camalexin, a well characterized phytoalexin, was attributed partly to its activity to disrupt bacterial membrane integrity [46]. The production of phytoalexins can be induced by either microbial infections or various abiotic treatments [44]. *Arabidopsis* camalexin production was induced by various virulent and avirulent *Pseudomonas syringae* strains [46,47]. In addition, highly conserved microbe-associated molecular patterns, such as bacterial flagellin, were shown to trigger camalexin production as part of the pattern triggered immunity [48]. The non-model crop plant canola (rapeseed) is one of the world’s most important oilseed crops. Analyses of metabolites from canola infected with the fungal phytopathogen *Plasmodiophora brassicae* (clubroot) or *Albugo candida* showed that canola responded to fungal pathogens by the production and accumulation of phytoalexins in their roots [49,51]. However, canola’s metabolomic response to bacterial pathogens has not been studied. Using a root infection system established in our laboratory to characterize the canola*–P. aeruginosa* interactions, we probed the chemical responses during the interaction of canola with the opportunistic bacterial pathogen *P. aeruginosa* strain PA14 in this study. We discovered that *P. aeruginosa*-secreted QS metabolites (both signalling molecules and regulated products) played an important role in the plant–microbe interaction based on the MS metabolomics results. We also demonstrated the induction of a plethora of phytoalexin production and exudation by canola over the course of the 5 day infection experiments.

## Methods

### Plant treatment and infection experiment

In this study, we used a root infection system of *P. aeruginosa* in liquid culture using the seedlings of an important oilseed crop, canola (*Brassica napus*), as previously described [52]. Briefly, canola seeds (Mumm's Sprouting Seeds) were surface sterilized and planted on MS medium [Murashige and Skoog basal medium with vitamins (Phytotechnology Laboratories) supplemented with 0.5 g MES hydrate l^−1^ and 0.5% sucrose at pH 5.7] agar plates. Seven-day-old seedlings were transferred to 50 ml conical tubes containing 5 ml MS liquid media (where only the roots were submerged in the MS media) and grown on a growth light stand (Hydrofarms) for 3 more days at 22 °C, under 16 h of daylight (750 lumens) before infection. An overnight culture of *P. aeruginosa* strain PA14 (WT), a primary clinical isolate from a burn patient [53,54], was washed twice using 10 mM MgSO_4_ and the bacterial optical density at 600 nm was adjusted to 0.1 (~1×10^8^ cells ml^−1^) in 5 ml MS medium for the infections. This bacterial inoculum amount was previously optimized in our laboratory (data not shown), and is within the range (1.6×10^7^–1×10^8^ cells ml^−1^) reported in the literature for *P. aeruginosa* root infections [55,56]. The bacterial inoculum was added to 10-day-old canola seedlings to replace the original MS medium in the conical tubes. Uninfected canola and bacterial inoculum without plants were included as controls. MS media from infected and uninfected seedlings and bacterial only cultures were collected on days 1, 3 and 5 post-infection. One hundred microlitres of the media was serially diluted and plated on LB agar plates for bacterial counting. The rest of the samples were spun down at 5 000 ***g*** for 10 min to remove bacteria from the samples (infected plants and bacterial control). The supernatants were stored at −20 °C for liquid chromatography-high resolution MS (LC-HRMS) analysis and metabolomics profiling. This experiment was done in four independent biological replicates.

For the infection with the QS mutants, WT PA14 and its isogenic mutants Δ*lasR* [57], Δ*lasI* [58], Δ*rhlR* [57], Δ*rhlI* [59] and Δ*mvfR* [60], were prepared as described above for the canola infection. One hundred microlitres of MS media from infected seedlings was collected on days 1, 3 and 5 post-infection for bacterial counting. At the end of the 5 day infection, the remaining MS media were spun down at 5 000 ***g*** for 10 min to remove bacteria from the samples. The supernatants were stored at −20 °C for LC-HRMS analysis and metabolomics profiling. This experiment was done in three independent biological replicates.

For the QS signalling molecule treatment experiments, 10 μM C4-HSL, 3-oxo-C12-HSL and PQS purchased from Sigma-Aldrich in 5 ml MS medium was added separately (10 mM stock solutions were prepared in DMSO) to 10-day-old canola seedlings to replace the original MS medium in the conical tubes. The same volume of DMSO solvent was used as a control. The concentration of these QS signalling molecules was chosen based on our absolute quantification of their concentrations in PA14 cultures. MS media from treated and untreated seedlings were collected on day 5 post-treatment for the subsequent LC-HRMS analysis. This experiment was done in three independent biological replicates.

### Metabolomics analysis

For the metabolomics analysis, 4 ml of the above-described MS media supernatants was cleaned up and eluted using 2% formic acid in methanol using a Bond Elut C18 column (Agilent). The samples were dried using a centrifugal evaporator, and then reconstituted in 100% methanol containing 1 µg reserpine ml^−1^ as an internal standard. The samples were analysed using a LC-HRMS system consisting of an UltiMate 3000 LC pump (Thermo Fisher Scientific) coupled to an Exactive high resolution mass spectrometer, equipped with an electrospray ionization source. Separation was carried out on an Acquity HSS T3 column (2.1×100 mm, 1.8 µm; Waters). The solvent comprised of (A) 0.1% formic acid in de-ionized water and (B) 0.1% formic acid in acetonitrile at 0.35 ml min^−1^, with a linear gradient from 5–100% B in 4 min. MS acquisitions were performed in positive polarity, with resolution of 25000 instrument settings. The ion source conditions consisted of spray voltage of 2.5 kV, sheath flow of 50, auxiliary flow of 10, with capillary and heater temperatures of 360 and 300 °C, respectively.

LC-HRMS raw data files were converted into netCDF files using a built-in module in the software XCalibur, and the resulting MS data were then processed in the open-source software mzMine 2 [61] to obtain binned or bucketed MS data suitable for multivariate statistical analysis. The processing steps include mass detection, chromatogram building, deisotoping and joint alignment to create a list of buckets defined with a variable ID, a retention time and mass to charge ratio (*m*/*z*). Peak areas attributed to each bucket for all the samples were then studied using partial least squares-discriminant analysis (PLS-DA; SIMPA-P+ 12.0.1; Umetrics) in order to highlight trends and discrepancies in the dataset and key metabolites. The samples were grouped into classes as day 1 bacterial control (D1-BC), day 1 plant control (D1-PC), day 1 infected (D1-Infected), day 3 bacterial control (D3-BC), day 3 plant control (D3-PC), day 3 infected (D3-Infected), day 5 bacterial control (D5-BC), day 5 plant control (D5-PC) and day 5 infected (D5-Infected). The putative identification of highlighted variables or metabolites was done based on the interpretation of the HRMS data including calculated molecular formula, isotopic pattern and literature search on SciFinder. Reference standards of pyocyanin, C4-HSL, 3-oxo-C12-HSL, PQS, HHQ and rhamnolipids were acquired from Sigma-Aldrich to identify key observable ions by matching retention time, accurate mass and MS/MS fragmentation patterns. The MS/MS data were acquired on a QTOF Premier quadrupole time-of-flight mass spectrometer (Waters) equipped with an electrospray ionization LockSpray modular source, using lock mass solution of leucine enkephalin (33 pg µl^−1^). Chromatographical separation conditions were the same as described above. Capillary voltage was at 3.2 kV, sample cone voltage at 32 V, collision energy at 30 eV, with source and desolvation temperatures set to 120 and 350 °C, respectively.

The relative abundances of metabolites were presented as peak area from extracted ions with mass accuracy within 5 p.p.m. (normalized using internal standard reserpine). Box plots were created using a web-based tool BoxPlotR (http://shiny.chemgrid.org/boxplotr/). The metabolite quantification experiment was done in four independent biological replicates with duplicate injections of each biological sample. Statistical analysis was performed on the peak area of each target metabolite or extracted ion chromatogram after normalization using reserpine as an internal standard using Student’s *t*-test. *P* values <0.05 were considered significant.

For samples from the infection with QS mutants and QS signalling molecule treatment, the same sample preparation procedures described above were used. LC-HRMS analysis was done using a high-resolution mass spectrometer Q Exactive (Thermo Fisher Scientific). The MS parameters were the same as previously described for the Exactive mass spectrometer, with resolution at 35000 and 17000 instrument settings, for MS and MS/MS, respectively, with collision energy at 30 eV. The Q Exactive was also used for confirming the identity and quantitation of brassilexin. The reference standard of brassilexin was purchased from Toronto Research Chemicals. The identification was done based on matched retention time, accurate mass and MS/MS fragmentation data between the standard and in a collected sample. Quantitation of brassilexin in growth media of infected and plant control samples was carried out using the same LC-HRMS instrument. Concentration was calculated based on the peak area of the extracted precursor ion and the calibration curve of the standard.

### Determination of the MIC of brassilexin on *P. aeruginosa* PA14

To determine the susceptibility of *P. aeruginosa* to phytoalexins, *P. aeruginosa* strain PA14 was incubated with the phytoalexin brassilexin to assess its antibacterial activity. Overnight cultures of *P. aeruginosa* strain PA14 were grown in LB broth for 20 h at 37°C. Same day cultures were prepared by diluting 100 µl of the overnight culture in 5 ml LB and were grown for 3 h to mid-log phase (an OD_600_ of approximately 0.5). Cell suspensions were then standardized through dilution in LB to an OD_600_ of 0.0002.

Brassilexin powder was dissolved in DMSO to a concentration of 10 mg ml^−1^. The antibacterial nature of brassilexin was determined using the microdilution method as described by the Clinical and Laboratory Standards Institute (CLSI). A set of brassilexin dilutions (concentration range from 800 to 6.125 µg ml^−1^) was prepared in 50 µl LB broth. After the dilutions were prepared, 50 µl bacterial suspension prepared as above was mixed with each dilution. The final volume of each well was 100 µl. A vehicle control with the DMSO was included as a comparison to brassilexin treatment. All of the treated and control samples were incubated on a shaker at 250 r.p.m. for 20 h at 37 °C. After incubation, the OD_600_ was measured to determine the growth of the samples. A total of four independent biological replicates were performed in technical duplicates. The data were analysed using Student’s two-tailed *t*-test and one-way ANOVA using Tukey’s test to compare mean separations of the measured optical density measurements for bacterial growth between treated samples and the vehicle control. Differences were considered statistically significant at *P*<0.05.

## Results

### Demonstration of canola-supported *P. aeruginosa* growth

Previously, we have established a root infection model to characterize canola*–P. aeruginosa* interactions [52]. We showed the colonization of *P. aeruginosa* strain PA14 to the roots of canola [52] and this colonization activity was decreased in the QS mutants [42]. In this work, we focus on the metabolomic profile changes during the canola*–P. aeruginosa* interactions. To begin with, we investigated whether there was active growth of *P. aeruginosa* during interactions with canola, compared to the medium alone. We serially diluted and plated bacteria from the PA14 grown in the absence (bacterial control; BC) and presence of canola samples (Infection). The bacteria controls throughout the whole 5 day period showed an identical count of approximately 1×10^8^ c.f.u. ml^−1^, which was approximately equal to the inoculum (Fig. S1, available with the online version of this article). However, the presence of plant hosts increased the bacterial count by about tenfold on day 1 post-infection and supported the stable bacterial counts afterwards (Fig. S1).

### Identification of metabolites shaping the *P. aeruginosa*–canola interaction

**Fig. 1.**
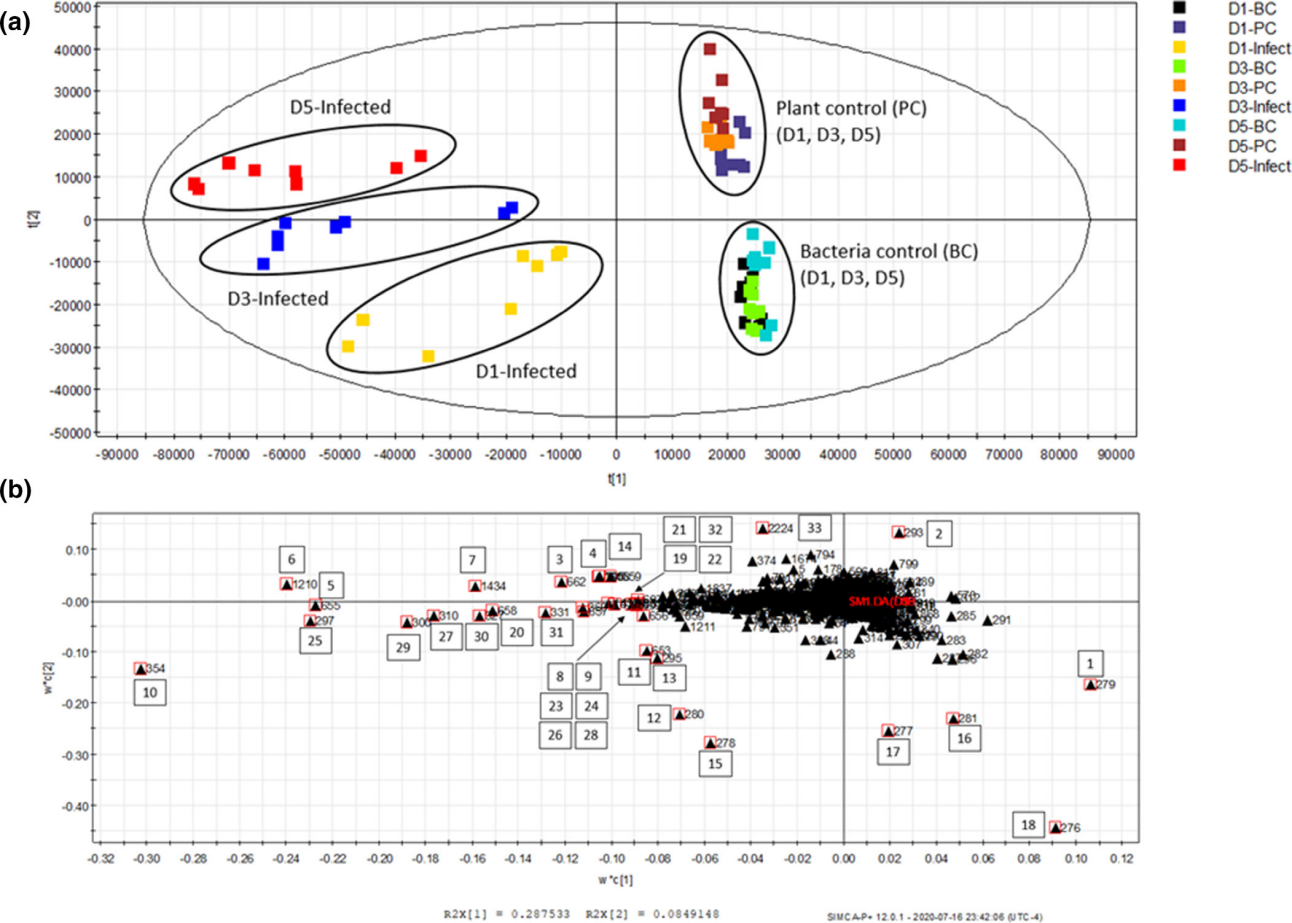
PLS-DA score and loadings plots obtained from the LC-MS data recorded for *P. aeruginosa* infected samples, bacterial control and plant control samples. (a) Score plot displaying clusters and trends in the LC-HRMS dataset. The data were processed using PLS-DA. The confidence interval of 95% was used for each component in the score plot. *n*=4 biological replicates with analytical duplicates for each. (b) Loadings plot revealing key variables (retention time and *m*/*z*) correlated to cluster and trends in the score plots. A number was assigned to each key variable listed in Table 1 to help with the description of the results in the paper. D1-Infect, D3-Infect and D5-Infect represent the samples on days 1, 3 and 5 post-*P*. *aeruginosa* infection, respectively. Similarly, D1-BC, D3-BC and D5-BC represent bacterial control samples, whereas plant control samples are identified as D1-PC, D3-PC and D5-PC.

LC-HRMS was chosen as a suitable analytical technique to capture the complex chemical compositions of the growth media resulting from canola seedling infected by *P. aeruginosa*. To highlight trends and discrepancies between experiments, untargeted metabolomics analyses, and in particular PLS-DA, were conducted. In this study, PLS-DA was used as a discovery tool to identify key variables or metabolites in the dataset that are involved in differentiating bacterial and plant controls from the *P. aeruginosa*-infected canola samples. As shown in Fig. 1a, the infected canola samples were found to distinctly cluster on the left-hand side of the PLS-DA score plot and away from either the bacteria or plant controls. These observations suggested the presence of unique variables or metabolites associated with *P. aeruginosa*–canola interactions. Interestingly, a trend of progression within the infected samples (D1-, D3- and D5-Infected) was also observed over 5 days, indicating that the metabolomics interactions were evolving during the course of the experiments. That is to say that the chemical profiles of the infected samples were becoming more distinct from those observed in both bacterial and plant control samples. The interpretation of the loadings plot (Fig. 1b) led us to highlight the key variables responsible for the clustering and discriminatory patterns displayed in the score plot (Fig. 1a). For LC-HRMS datasets, variables correspond to binned MS data defined by a retention time and a mass to charge ratio (*m*/*z*), and the key variables highlighted by PLS-DA are listed in Table 1. The location of the variables or pseudomolecular ions on the loadings plot can be directly correlated to key trends in the experimental results including the separation of the samples into each experimental group (e.g. bacterial control, canola plant control, days 1, 3, and 5 infected). For example, ion numbers 5, 6, 10 and 25 displayed on the left-hand side of the loadings plot were expected to be more abundant in *P. aeruginosa*-infected canola samples, while the ion numbers 16, 17 and 18 are features attributed to the bacterial control samples.

**Table 1.** List of pseudomolecular ions with higher discriminative power (as shown in Fig. 1b) in untargeted metabolomic analysis using PLS-DA

Ion no.	Variable ID	Retention time (min)	Observed accurate mass (*m*/*z*)	Molecular formula	Calculated mass	Mass error (p.p.m.)	Tentative metabolite annotation [reference]
1	279	0.70	365.10520	C_12_H_22_O_11_	365.10543[M+Na]^+^	−0.63	Sucrose
2	293	0.71	218.04546	Unknown	–	–	–
3	662	2.71	160.07571	C_10_H_9_NO	160.07569[M+H]^+^	0.12	Indole-3-acetaldehyde [62]
4	795	2.74	496.24447	C_27_H_33_N_3_O_6_	496.24421[M+H]^+^	0.52	Cyclic spermidine conjugate [82]
5	655	3.56	224.08181	C_13_H_9_N_3_O	224.08184 [M+H]^+^	−0.13	Phenazine-1-carboxamide (PCN)
6	1210	3.86	313.35767	Unknown	–	–	–
7	1434	3.86	177.69576	Unknown	–	–	–
8	1433	4.04	339.37337	Unknown	–	–	–
9	656	4.04	207.05531	C_13_H_7_N_2_Ofragment ion	207.05529	0.10	Phenazine-1-carboxylic acid (PCA) fragment (loss of H_2_O)
10	354	4.05	225.06573	C_13_H_8_N_2_O_2_	225.06585 [M+H]^+^	−0.53	Phenazine-1-carboxylic acid (PCA)
11	653	4.09	226.07482	Unknown	–	–	Isotope peak of PCA
12	280	4.15	244.16899	C_16_H_21_NO	244.16959 [M+H]^+^	−2.46	4-Hydroxy-2-heptylquinoline (HHQ)
13	295	4.15	260.16500	C_16_H_21_NO_2_	260.16451 [M+H]^+^	1.88	2-Heptyl-4-hydroxyquinoline N-oxide (HQNO) [83]
14	1808	4.17	239.08138	C_14_H_10_N_2_O_2_	239.08150[M+H]^+^	−0.50	5-Methyl-phenazine-1-carboxylic acid [84]
15	278	4.18	325.06760	C_14_H_16_N_2_O_3_S_2_	325.06751 [M+H]^+^	0.28	Pyochelin
16	281	4.56	270.18522	C_18_H_23_NO	270.18524 [M+H]^+^	−0.07	4-Hydroxy-2-nonenylquinoline (or isomer) [25]
17	277	4.70	270.18524	C_18_H_23_NO	270.18524 [M+H]^+^	0	4-Hydroxy-2-nonenylquinoline (or isomer) [25]
18	276	4.73	272.20080	C_18_H_25_NO	272.20089 [M+H]^+^	−0.33	4-Hydroxy-2-nonylquinoline (HNQ) [25]
19	683	4.98	239.08147	C_14_H_10_N_2_O_2_	239.08150[M+H]^+^	−0.13	1-Carbomethoxyphenazine
20	658	5.00	240.08934	Unknown	–	–	–
21	1559	5.00	240.08925	Unknown	–	–	–
22	671	5.26	153.12738	C_10_H_17_Ofragment ion	153.12739	−0.07	Rhamnolipid RL2 fragment
23	360	5.26	171.13796	C_10_H_19_O_2_fragment ion	171.13796	0	Rhamnolipid RL2 fragment
24	517	5.26	293.12308	C_12_H_21_O_8_fragment ion	293.12309	−0.03	Rhamnolipid RL2 fragment (di-rhamnose)
25	297	5.27	668.42219	C_32_H_58_O_13_	668.42157 [M+NH_4_]^+^	0.93	Rhamnolipid RL2 (Rha- C_10_-C_10_)
26	657	5.27	673.37756	C_32_H_58_O_13_	673.37696[M+Na]^+^	0.89	Rhamnolipid RL2 (Rha- C_10_-C_10_)
27	310	5.27	359.27923	C_20_H_39_O_5_fragment ion	359.27920	0.08	Rhamnolipid RL2 fragment (loss of 2 rhamnose units)
28	699	5.60	694.43760	C_34_H_60_O_13_	694.43722 [M+NH_4_]^+^	0.55	Rhamnolipid MW676 (Rha-Rha-C_10_-C_12_, unsaturated)
29	300	5.64	522.36412	C_26_H_48_O_9_	522.36366 [M+NH_4_]^+^	0.88	Rhamnolipid RL1 (Rha-C_10_)
30	327	5.64	527.31977	C_26_H_48_O_9_	527.31905 [M+Na]^+^	1.37	Rhamnolipid RL1 (Rha-C_10_)
31	331	5.64	359.27922	C_20_H_39_O_5_fragment ion	359.27920	0.06	Rhamnolipid RL1 fragment (loss of rhamnose)
32	697	5.85	696.45358	C_34_H_62_O_13_	696.45287 [M+NH_4_]^+^	1.02	Rhamnolipid MW678 (Rha-Rha-C_10_-C_12_)
33	2224	7.05	537.53578	Unknown	–	–	–

The putative identification of the metabolites related to the key variables (retention time and *m*/*z*) observed by PLS-DA (see Table 1) was attempted using a multi-step approach. Firstly, the pseudomolecular ions (*m*/*z*) were examined to identify the adduct ions (e.g. MNa^+^, MK^+^ and MNH_4_^+^). Then, all possible molecular formulae were calculated in the XCalibur software using a mass accuracy within 5 p.p.m. Based on the isotopic pattern, the most probable molecular formulae were finally searched in SciFinder, a literature and chemical database from the American Chemical Society and matched with putative metabolites related to plant- or bacterial-based natural products. As a result, 17 metabolites were putatively identified, accounting for 26 variable IDs listed in Table 1.

Among the metabolites examined, the majority of them were identified as being secreted by *P. aeruginosa*, including five quinolones (ion numbers 12, 13, 16–18), four phenazines (ion numbers 5, 10, 14, 19), four rhamnolipids (ion numbers 25, 28, 29, 32 that represent the major parental ions, while all ions, including the minor derivatives, are included in Table 1 as ion numbers 22 through 32), and the siderophore pyochelin (ion number 15). It appears that indole-3-acetaldehyde (ion number 3) and cyclic spermidine conjugate (ion number 4) are the only two plant-derived compounds identified as key variables in the PLS-DA plots. This is most likely explained by the overabundance of bacterial metabolites secreted in the growth media when compared to the plant root exudates. Indole-3-acetaldehyde (ion number 3) was detected in the growth medium and is known to be a precursor to indole-3-acetic acid (IAA), a plant hormone auxin present in canola [62]. However, *Pseudomonas syringae* pv. *tomato* strain DC3000 was also shown to use indole-3-acetaldehyde as a substrate of the enzyme indole-3-acetaldehyde dehydrogenase AldA to produce IAA, as a virulence strategy to suppress salicylic acid-mediated defences in *Arabidopsis thaliana* [63]. Many strains of pseudomonads, including *P. aeruginosa*, have genes encoding proteins with ~90–95% sequence identity to AldA and can produce indole-3-acetaldehyde and IAA [63]. Nevertheless, the indole-3-acetaldehyde (ion number 3) was only detectable in the infected and plant control samples, and the amount was below the detection level in the bacteria only controls at all time points. Similarly, although *P. aeruginosa* genome encodes spermidine biosynthesis genes [64], cyclic spermidine conjugate (ion number 4) was only detectable when canola was present. Therefore, the plant-associated metabolites, indole-3-acetaldehyde and cyclic spermidine conjugate, detected in our untargeted analysis were most likely a product from canola; however, they could not be completely ruled out as *P. aeruginosa*-produced metabolites.

### Targeted analysis of QS molecules and other metabolites

While the untargeted approach above led to the identification of a number of discriminative ions, targeted analysis was also explored to assess other plant–bacterial interaction-associated metabolites. Quinolone HHQ (ion number 12) is a key *P. aeruginosa* QS signalling molecule, and its identification by PLS-DA led us to further investigate other QS signalling or associated metabolites. Consequently, the reference standards C4-HSL, 3-oxo-C12-HSL, PQS and pyocyanin were acquired and their LC-HRMS features (retention time and observed accurate mass) were recorded using the same experimental conditions (see Table 2). In a similar manner, the identity of HHQ (ion number 12) and the rhamnolipids (ion numbers 22–32) were confirmed using their respective commercially available standards HHQ, rhamnolipid RL1 and rhamnolipid RL2. Their matching retention times, observed accurate masses and MS/MS fragmentation patterns are illustrated in the supporting materials (Fig. S2).

**Table 2.** List of QS-related metabolites detected in targeted metabolomics analysis

Metabolite	Retention time (min)	Observed accurate mass (*m*/*z*)	Molecular formula	Calculated mass (*m*/*z*) [M+H]^+^	Mass error (p.p.m.)
C4-HSL	2.41	172.09659	C_8_H_13_NO_3_	172.09682	−1.34
3-Oxo-C12-HSL	2.79	298.20123	C_16_H_27_NO_4_	298.20129	−0.20
PQS	4.45	260.16400	C_16_H_21_NO_2_	260.16451	−1.96
Pyocyanin	2.25	211.08670	C_13_H_10_N_2_O	211.08659	0.52

**Fig. 2.**
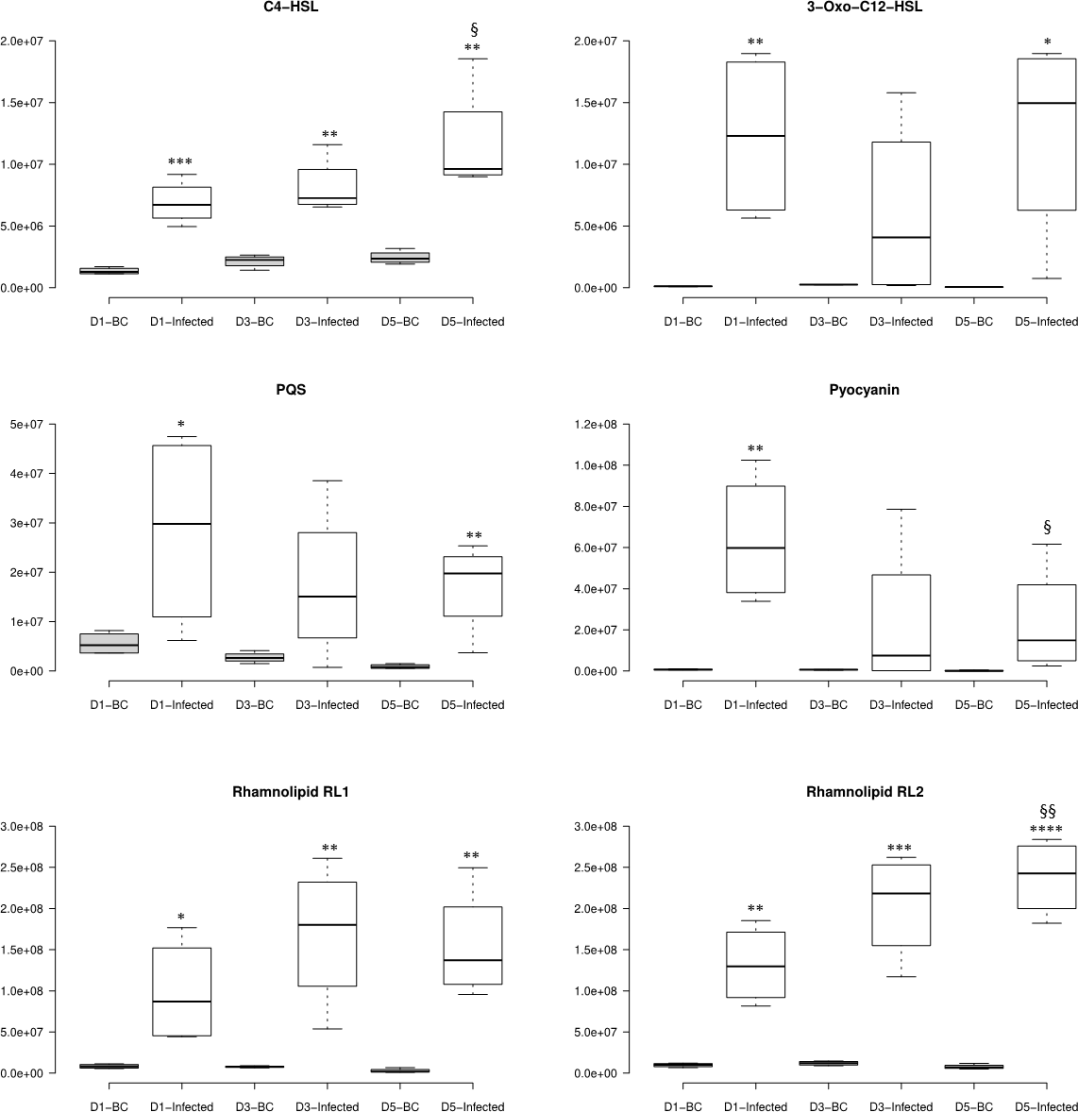
Levels of key selected QS signalling and associated metabolites in growth media of canola seedling with *P. aeruginosa* infection. The *y*-axes display the peak area of each target metabolite normalized using reserpine as an internal standard. Centre lines on each box plot show the medians; box limits indicate the 25th and 75th percentiles as determined by R software; whiskers extend 1.5 times the interquartile range from the 25th and 75th percentiles, outliers are represented by dots. *n*=4, which includes four biological replicates, each with duplicated LC-MS analysis. D1, D3 and D5 represent the samples on days 1, 3 and 5 post-*P*. *aeruginosa* infection, respectively. BC, bacterial control. Infected, canola with *P. aeruginosa* infection. Metabolite levels were assessed by Student’s *t*-test. *, compared with BC; §, compared with D1-infected sample. * or §, *P*<0.05; ** or §§, *P*<0.01; ***, *P*<0.0001; ****, *P*<0.00001.

Little is known about the dynamics of QS-associated-metabolite production by *P. aeruginosa* in the presence of a plant host. In order to assess the dynamic changes of their concentrations in the culture media, the LC-HRMS data were further processed to integrate the peak areas of the extracted ion chromatograms using a mass accuracy <5 p.p.m. for each of these metabolites over the course of the 5 day *P*. *aeruginosa* infection experiment. The plant hosts supported more PA14 growth compared to the bacterial controls (Fig. S1); therefore, it is not surprising that the peak areas of all the QS-associated metabolites (normalized to the same volume of plant growth media) were higher in the samples with plant hosts (Infected) compared to the bacterial controls (BCs) (Fig. 2). To identify the impact of canola on the production of QS-associated metabolites and eliminate the interference of the bacterial abundance in the media, we also presented the relative quantification of the QS-associated molecule data based on the normalization to bacterial counts (Table 3). However, it is obvious that even if normalizing to bacterial counts, the differences in QS metabolites still showed an increased trend between the Infected versus BC samples (with the exception of C4-HSL), suggesting the higher bacterial metabolites were not only a reflection of the bacterial abundance in the media but also due to the dynamics of an infection of canola. Therefore, we focus on the comparison below using the data normalized to the media volume.

**Table 3.** QS-related metabolites area normalized to bacterial counts (peak areas of the same metabolites were normalized to media volume as shown in Fig. 2) The numbers represent the mean (four biological replicates) peak area of each target metabolite normalized to bacterial count ± sd. D1, D3 and D5 represent the samples on days 1, 3 and 5 post-*P. aeruginosa* infection, respectively. BC, bacterial control. Infected, canola with *P. aeruginosa* infection.

Metabolite	D1 BC	D1 infected	D3 BC	D3 infected	D5 BC	D5 infected
C4-HSL	0.014±0.003	0.007±0.002	0.022±0.005	0.008±0.002	0.025±0.005	0.012±0.005
3-Oxo-C12-HSL	0.001±0.0002	0.012±0.001	0.003±0.0001	0.006±0.007	0.0005±0.0001	0.013±0.009
PQS	0.057±0.023	0.028±0.02	0.028±0.011	0.017±0.0015	0.009±0.004	0.017±0.01
Pyocyanin	0.007±0.002	0.064±0.032	0.006±0.002	0.023±0.037	0.002±0.002	0.024±0.027
Rhamnolipid RL1	0.085±0.027	0.099±0.065	0.078±0.01	0.165±0.085	0.029±0.026	0.157±0.068
Rhamnolipid RL2	0.101±0.025	0.132±0.048	0.121±0.026	0.199±0.063	0.076±0.029	0.241±0.047

As shown in Fig. 2, the two classes of key QS signalling molecules, the AHLs (C4-HSL and 3-oxo-C12-HSL) and quinolone (PQS), were all higher in canola growth media with *P. aeruginosa* root infection. Pyocyanin, a *P. aeruginosa* phenazine, was also observed at a much higher level upon interacting with canola. This virulence factor, produced in response to PQS signalling, had been elucidated as the terminal physiological signal in the QS network [65]. In addition, rhamnolipids (RL1 and RL2) were found to be significantly higher (>10-fold) in samples with canola growing with the *P. aeruginosa* infection. RLs are QS-controlled metabolites, which are important to the maintenance and functions of biofilm [66], were shown to have direct effects on plant defence against fungal infection [67,68].

Similarly, the relative abundance of the other metabolites identified in Table 1 were monitored over the course of the 5-day experiment. As shown in Fig. S3 (-1 to -5), in the presence or absence of canola from 1 to 5 days, quinolone levels in the growth media of *P. aeruginosa* did not follow the same trend within this group of structurally similar metabolites. Similar to the trends for PQS, HHQ (ion number 12) and 2-heptyl-4-hydroxyquinoline N-oxide (HQNO, ion number 13) levels were higher in the presence of canola host compared to the bacterial control. However, the levels of 4-hydroxy-2-nonenylquinoline (ion number 16) and its isomer (ion number 17), and 4-hydroxy-2-nonylquinoline (HNQ, ion number 18), were lower in infection samples compared to bacterial control.

Similar to pyocyanin, other phenazines such as phenazine-1-carboxamide (PCN, ion number 5), phenazine-1-carboxylic acid (PCA, ion number 10) and 1-carbomethoxy-phenazine (ion number 19) were found to be higher in media with canola seedling over the 5 days post-infection, though PCA trended down while others gradually increased. 5-Methyl-phenazine-1-carboxylic acid (ion number 14) appeared to be only present in media with canola starting on day 3 and the level went up further on day 5. PCA is a known precursor of other phenazines including PCN and pyocyanin [65], so it was likely partially consumed over time as its level had dropped (Fig. S3-6 to S3-9).

Other *P. aeruginosa* metabolites tentatively identified including two additional rhamnolipids coded as RL MW676 (ion number 28) and RL MW678 (ion number 32), and pyochelin (ion number 15), were at higher levels in media with canola on day 1 after infection, compared to the bacterial control (Fig. S3-10 to -12). It appeared that these RLs exhibited a similar trend as the two standard-verified analogues RL1 and RL2, with sustained or even increasing production on days 3 and 5. Pyochelin, however, gradually dropped to similar or even lower levels than the bacterial control after day 1. Clearly, upon interaction with canola and its root exudates, *P. aeruginosa* continued to produce increasingly higher levels of QS signalling metabolites, presumably contributing to mediating the interactions with its plant host.

### Root-exuded metabolites from *Brassica napus*

Untargeted metabolomics revealed little information on *Brassica napus* root-exuded metabolites upon *P. aeruginosa* infection, possibly due to the rapid growth of the bacteria leading to much higher levels of its metabolites present in the media. Nevertheless, two seemingly plant-associated metabolites, indole-3-acetaldehyde (ion number 3) and cyclic spermidine conjugate (ion number 4) were putatively identified by PLS-DA as indicated in Table 1. Their levels in the growth media were higher in the infected samples as compared to plant controls and were trending up over the 5 days in the infected samples (Fig. S4-1 and -2).

The synthesis of plant defence metabolites such as phytoalexins is controlled by auxin, and elevated levels of IAA and associated phytoalexins were reported in plants during pathogen infection [69]. With indole-3-acetaldehyde, a precursor of IAA, as the lead in the context of plant–microbe interaction, we attempted to examine phytoalexin production in canola upon *P. aeruginosa* infection. Therefore, the LC-HRMS data were queried for a list of phytoalexins that have been reported in the literature and identified in cruciferous plants. The theoretical accurate mass was calculated for the adduct ion [M+H]^+^ and the corresponding extracted ion chromatograms were monitored using a mass accuracy within 5 p.p.m. The lead pseudomolecular ions were further confirmed by matching the isotopic pattern, which led to the identification of eight putative phytoalexins observed in the *P. aeruginosa* infected samples (Table 4).

**Table 4.** List of phytoalexin-like ions detected in the LC-HRMS data by targeted analysis

Ion no.	Retention time (min)	Observed accurate mass (*m*/*z*)	Molecular formula	Calculated mass[M+H]^+^	Mass error (p.p.m.)	Other associated ion observed (*m*/*z*)	Tentative phytoalexin [reference]
34	2.84	479.07999	C_17_H_22_N_2_O_10_S_2_	479.07886	2.36	Fragment ions399.12265 (C_17_H_23_O_7_N_2_S)237.06943(C_11_H_13_O_2_N_2_S)205.04301 (C_10_H_9_N_2_OS)187.08658 (C_11_H_10_N_2_O)	4-Methoxyglucobrassicin (MGB) [51]
35	3.84	192.04799	C_10_H_9_NOS	192.04776	1.20	–	Brassicanal A [51]
36	3.96	175.03258	C_9_H_6_N_2_S	175.03244	0.80	–	Brassilexin [51]
37	3.97	251.03061	C_11_H_10_N_2_OS_2_	251.03073	−0.48	–	Spirobrassinin [51]
38	3.99	176.07068	C_10_H_9_NO_2_	176.07060	−0.45	Fragment ion133.05244 (C_8_H_7_ON)	1-Methoxy-3-indolecarboxaldehyde (MICA) [51]
39	4.15	265.04630	C_12_H_12_N_2_OS_2_	265.04638	−0.30	Fragment ion 233.01953 (C_11_H_9_N_2_S_2_)	4-Methoxycyclobrassinin [51]
40	4.38	265.04648	C_12_H_12_N_2_OS_2_	265.04638	0.38	Fragment ions233.02042 (C_11_H_9_N_2_S_2_)187.08658, C_11_H_10_N_2_O	Sinalbin B [51]
41	4.91	295.05731	C_13_H_14_N_2_O_2_S_2_	295.05694	1.25	–	Wasalexin A or B [49]

After normalization with the internal standard reserpine, the levels of the eight phytoalexins based on their peak area, on days 1, 3 and 5 after infection, were monitored. The results are summarized in Fig. S4-3 to -10. In comparison to canola control, *P. aeruginosa* infection was found to induce the production and secretion of 4-methoxyglucobrassicin (MGB, ion number 34), brassicanal A (ion number 35) and brassilexin (ion number 36). MGB appeared to be induced very rapidly, as it was higher on day 1 and then slowly dropped. Brassicanal A and brassilexin, however, only showed up on day 3 with their highest observed level reached on day 5. These three phytoalexins were not present in the canola control. However, spirobrassinin (ion number 37), 1-methoxy-3-indolecarboxaldehyde (MICA; ion number 38), 4-methoxycyclobrassinin (ion number 39) and sinalbin B (ion number 40) were detected in trace amounts in the canola control, while they were greatly boosted during *P. aeruginosa* infection, particularly on days 3 and 5. Canola seemed to exude and release wasalexin A or its isomer B (ion number 41) on its own, and the bacterial infection further elicited its production. Its level, however, was not changed that much over the 5 days.

### QS-dependent and -independent induction of phytoalexins by *P. aeruginosa* infection

Because the QS molecules were identified in abundance and the phytoalexins were induced during the canola*–P. aeruginosa* interaction, we set out to examine whether the QS systems were responsible for the observed phytoalexin induction. First, we added the commercially available *P. aeruginosa* QS signalling molecules (C4-HSL, 3-oxo-C12-HSL and PQS at 10 µM) to the canola growth media for a period of 5 days. The concentration of these QS signalling molecules was chosen based on our absolute quantification of their concentrations in PA14 cultures (data not shown). After 5 days of treatment, none of these pure QS signalling molecules induced the phytoalexin production (data not shown), although their presence in the plant growth media was successfully detected and confirmed by MS data.

**Fig. 3.**
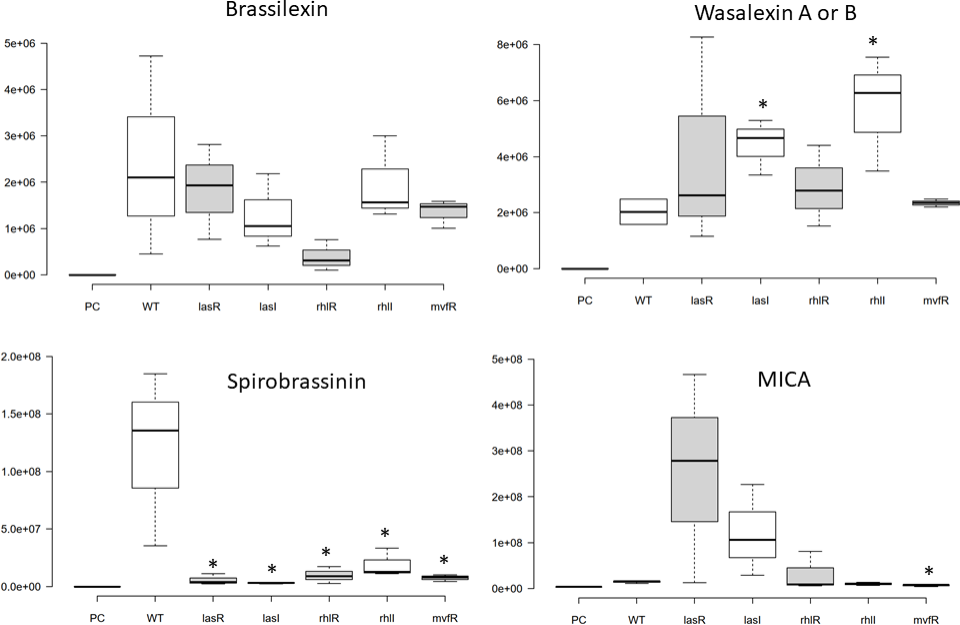
Levels of key selected phytoalexins in the growth media of canola seedlings with *P. aeruginosa* WT PA14 and QS mutant infections. The *y*-axes display the peak area of each target metabolite normalized using reserpine as an internal standard. Centre lines on each box plot show the medians; box limits indicate the 25th and 75th percentiles as determined by R software; whiskers extend 1.5 times the interquartile range from the 25th and 75th percentiles, outliers are represented by dots. *n*=3, which includes three biological replicates. PC, canola plant control; WT, wild-type PA14; *lasR*, *lasI*, *rhlR*, *rhlI* and *mvfR* represent the isogenic mutants of those genes in the PA14 background. Metabolite levels were compared to the WT and assessed by Student’s *t*-test. *, *P*<0.05.

Then, we used a collection of PA14 isogenic QS mutants that are abrogated in signalling in various QS branches, including Δ*lasR*, Δ*lasI*, Δ*rhlR*, Δ*rhlI* and Δ*mvfR*, in the infection of canola side-by-side with the WT PA14. Because the bacterial counts for all these mutant strains reached the same level of growth as WT in the media at 1×10^8^ c.f.u. ml^−1^ from day 1 to 5, as shown in Fig. S1, we took the same peak area normalization strategy (to media volume) for the phytoalexin induction analysis by various strains of PA14 on day 5 post-infection. As shown in Fig. 3, the effects of lacking specific QS signalling branches on the induction of selected phytoalexins varies. For brassilexin (ion number 36) and wasalexin A/B (ion number 41), all QS mutants showed a similar level of induction with the WT, except that Δ*rhlR* mutant showed a lower level of brassilexin induction (not significant, *P*=0.09), and Δ*lasI* and Δ*rhlI* mutants significantly enhanced the wasalexin A/B induction more than the WT. For spirobrassinin (ion number 37), all QS mutants showed a drastically and significantly reduced induction than the WT. Interestingly, the mutants from the LasRI branch of the QS system (especially the Δ*lasR* mutant), but not the others, demonstrated a much stronger, although not statistically significant (*P*=0.07), ability to induce the level of MICA (ion number 38). However, the Δ*mvfR* mutant induced significantly lower amounts of MICA compared to WT.

### Quantification and antibacterial activity of brassilexin

**Fig. 4.**
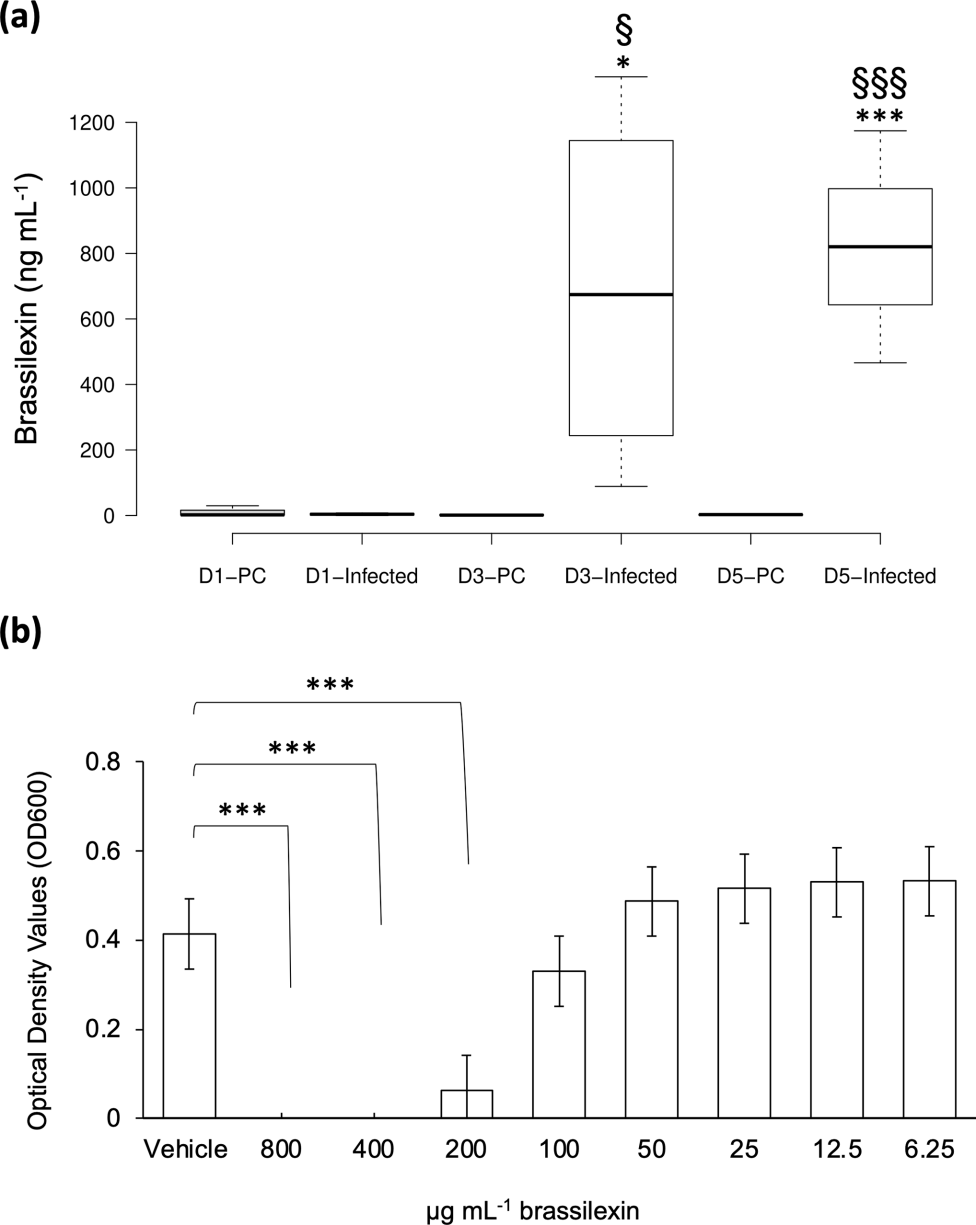
Quantification and antibacterial activity of brassilexin. (a) The *y*-axis displays the brassilexin levels in growth media calculated based on the standard curve constructed using the reference standard. Centre lines on each box plot show the medians; box limits indicate the 25th and 75th percentiles as determined by R software; whiskers extend 1.5 times the interquartile range from the 25th and 75th percentiles, outliers are represented by dots. *n*=4, which includes four biological replicates, each with duplicated LC-MS analysis. D1, D3 and D5 represent the samples on days 1, 3 and 5 post-*P*. *aeruginosa* infection, respectively. PC, plant control. Infected, canola with *P. aeruginosa* infection. Metabolite levels were assessed by Student’s *t*-test. *, compared with PC; §, compared with D1-infected sample. * or §, *P*<0.05; *** or §§§, *P*<0.0001. (b) Brassilexin produced significant inhibition of growth of *P. aeruginosa* PA14, beginning at 200 µg ml^−1^. Complete inhibition of growth was observed at 400 µg ml^−1^. *n*=4, which includes four independent biological replicates, each with duplicated technical replicates. The data were analysed using Student’s two-tailed *t*-test and one-way ANOVA using Tukey’s test to compare mean separations of the optical density measurements for bacterial growth between treated samples and the vehicle control. ***, *P*<0.001.

As brassilexin was the only detected phytoalexin available commercially, its identity was confirmed in growth media samples based on LC-HRMS and MS/MS fragments. MS/MS fragments for brassilexin observed in the infected samples were identical to the ones of the standard (Fig. S5). The concentrations of brassilexin in media of canola infected with PA14 as well as the plant control over 5 days were measured using LC-HRMS methodology. As shown in Fig. 4a, the brassilexin level was significantly increased on days 3 and 5 in the infected canola samples. The induction reached a median concentration of 820.25 ng ml^−1^ for the infected and 2.67 ng ml^−1^ for the plant control on day 5.

To evaluate the antibacterial potential of brassilexin, a microtitre-plate-based antibacterial assay was performed, and bacterial growth was monitored by measuring the optical density of the samples at 600 nm after 20 h exposure to brassilexin. Fig. 4b displays the antibacterial activity of brassilexin against *P. aeruginosa* PA14. It was found that brassilexin completely inhibited *P. aeruginosa* growth at a MIC concentration of 400 µg ml^−1^ across four biological replicates.

## Discussion

To our knowledge, this work is the first study to directly demonstrate a unique and dynamic metabolomics profile that was shaped by the presence of both plant host and the opportunistic bacterial pathogen *P. aeruginosa* (Fig. 1). Key ions driving the interactions between canola and *P. aeruginosa* during the course of a 5 day infection generated from the untargeted metabolomics analysis were putatively identified based on their LC-MS features (Table 1) including retention times, calculated molecular formulae, isotopic pattern and ion fragmentations. Most of these induced metabolites observed in the PLS-DA plots were attributed to a bacterial metabolic response, while one plant-based metabolite (cyclic spermidine conjugate, ion number 4) and one other plant hormone precursor (indole-3-acetaldehyde, ion number 3) originated from canola. It is worth noting that for 7 out of the top 33 key ions, the tentative identification based on accurate mass was not successful (Table 1). In addition, the identities of many other ions in the profiles could not be assigned (data not shown), suggesting the potential presence of novel metabolites that are involved in *P. aeruginosa*–canola interactions.

The bacterially synthesized products identified in the untargeted metabolomics belong to three major groups, quinolones, phenazines and rhamnolipids, all of which are involved in the *P. aeruginosa* QS processes. Therefore, we took a targeted search to look for other QS-associated molecules (Table 2). We then confirmed the identification of QS signalling molecules (C4-HSL and 3-oxo-C12-HSL from the RhlRI and LasRI AHL branches, and HHQ and PQS from the quinolone signalling branch) and QS-regulated metabolites (e.g. phenazine and rhamnolipid plus their derivatives) by comparisons using available standards (Fig. S2).

Furthermore, we compared the relative levels of these metabolites on days 1, 3 and 5 post-infection in the bacteria and plant only controls as well as the infected samples (Fig. 2). Our data demonstrated a clear overproduction of these QS-related molecules by *P. aeruginosa* in association with plants, suggesting an important role of the *P. aeruginosa* QS systems in mediating its interactions with plant hosts. Interestingly, *P. aeruginosa* quinolones, including the two major signalling molecules HHQ and PQS from the quinolone QS branch, represent the largest group of ions that shaped the interaction profiles. In our interaction study, there are other species of quinolone molecules (nonylquinolones) detected and the presence of plant hosts seemed to reduce their abundances, as they were shown to be at higher levels in the bacterial control (Fig. S3).

The two major detected QS-regulated metabolites (phenazine and rhamnolipid plus their derivatives) have been shown to be involved in the complex interactions with other soil microbes, including bacteria and fungi. For example, various phenazine derivatives secreted by other species of soil pseudomonads have been shown to be important for antagonistic interactions with fungal phytopathogens, such as *Gaeumannomyces graminis* [70,71] and *Fusarium oxysporum* [72], which causes the Take-all disease in wheat and barley, and tomato root rot, respectively. Rhamnolipids secreted by *P. aeruginosa* were shown to both trigger a protective host immunity in *Brassica napus* foliar tissues toward *Botrytis cinerea* and exert a direct antagonistic effect on this fungal pathogen [68]. Our work identified the production of QS-regulated phenazines and rhamnolipids by *P. aeruginosa* in association with plants, suggesting the QS systems can potentially play an important role in indirectly regulating plant growth and health via shaping plant microbiota in natural soils.

On the host side, plant metabolites play an important role in defence responses to pathogens [43]. However, little is known about plant metabolic response during the interaction with *P. aeruginosa*. Our initial untargeted metabolomics analysis identified one of the driving ions for the canola*–P. aeruginosa* interaction as the precursor of the plant hormone IAA, which is associated with phytoalexin production. This finding prompted us to use a targeted approach to further search the metabolomics profiles and identified eight phytoalexins (Table 4), all of which were induced by the *P. aeruginosa* infection. The basal levels for some of them [MGB (ion number 34), brassicanal A (ion number 35) and brassilexin (ion number 36)] were not detectable in the plant-only controls and required bacterial presence to be above the detection threshold (Fig. S4). All these identified phytoalexins from canola have demonstrated antibacterial or antifungal activities [73,74]. The induction of these phytoalexins in canola by *P. aeruginosa* suggests that plant hosts overproduce these compounds as a defence response to *P. aeruginosa* infection. The induced production of these phytoalexins happened very rapidly after bacterial treatment, with obvious increases on day 1 post-infection. With the exception of MGB (ion number 34), MICA (ion number 38) and wasalexin A/B (ion number 41), the other five phytoalexins showed incremental inductions that peaked on day 5 post-infection (Fig. S4).

To our knowledge, this is the first study to show *P. aeruginosa*-elicited phytoalexin production and exudation by plant roots, suggesting a role of these plant secondary metabolites in mediating the interaction between plants and this unique bacterial pathogen. The biosynthetic pathways of some phytoalexins (i.e. camalexin) in *Arabidopsis* [45,46, 75,78] and how they are suppressed by *Pseudomonas syringae* phytotoxin coronatine, an Ile-jasmonic acid mimic [48], have been well characterized. However, their biosynthesis pathways in canola remain largely unknown. Consistent with our finding that phytoalexins were overproduced in canola infected with *P. aeruginosa*, previous studies had shown that both conserved flagellin [79,80] and unique elicitors, such as a lysyl class protease (protease IV, PrpL) of *P. aeruginosa*, were able to induce camalexin biosynthetic genes in *Arabidopsis* [14]. In addition, a recent work of ours investigating the transcriptomic response of canola to *P. aeruginosa* infection had revealed that camalexin biosynthetic genes were highly induced over a 5 day infection course [52]. Although our previous study showed mild disease symptoms, including shoot and root weight losses, caused by *P. aeruginosa* using this root infection model [52], the metabolite data suggest the canola*–P. aeruginosa* interaction in natural soils can be reciprocal, where an opportunistic pathogenic bacterium primes the plant’s immune system to combat any other pathogens it encounters naturally, while the bacterium benefits from the nutrients and metabolites exuded by the plant.

A previous study has shown that AHLs ranging from 4 to 14 carbons in length, particularly C10-HSL, altered post-embryonic root development in *Arabidopsis* [81], which is a common effect of plant immunity elicitors [41]. The high abundance of QS signalling molecules detected in the presence of canola and the induction of plant-defence-related phytoalexins by *P. aeruginosa* infection prompted us to investigate whether *P. aeruginosa* QS systems were involved in the phytoalexin induction. The treatments of canola by commercially available QS signalling molecules, including C4-HSL, C12-HSL and PQS, did not increase phytoalexin production, suggesting that an active *P. aeruginosa* infection, instead of QS molecules, is required for the phytoalexin induction.

To further dissect the potential roles of each of the QS branches of *P. aeruginosa* in phytoalexin production, we used the QS mutants in the PA14 genetic background, including Δ*lasR*, Δ*lasI*, Δ*rhlR*, Δ*rhlI* and Δ*mvfR*, to infect canola. On days 1, 3 and 5 post-infection, bacterial counting from the media was performed for both bacteria grown with or without plants. There was no difference in growth for any the QS mutants, comparing to the ones observed for the WT (Fig. S1). This could be due to the unique root infection model that we used for this study. Unlike a previous study that introduced the WT and QS mutant (*lasI*^−^*rhlI*^−^) of *P. aeruginosa* strain PAO1 by coating germinated mung bean seedlings with bacterial suspension and focused on bacterial attachment to plant roots [41], the bacterial counting of the media in our study was more an indication of plant-exuded nutrients supporting bacterial growth rather than the reflection of virulence. Moreover, our recent publication that focused on the bacterial growth associated with canola root also showed slight but significant decrease in root colonization by all QS mutants compared to WT PA14 [42]. Interestingly, different QS mutants showed diverse impacts on the induction of various phytoalexins (Fig. 3). None of the mutants showed a significant difference in brassilexin induction, while all mutants showed dramatic and significant reduction in spirobrassinin induction, compared to the WT. Whereas, only certain mutants showed enhanced induction of wasalexin A/B and MICA. Taken together with the pure QS molecule treatment data, it seemed that QS-regulated bacterial effectors, instead of the QS signalling molecules themselves, were involved in regulating the phytoalexin induction during active infections. Similar to their complex and intertwined regulatory roles in *P. aeruginosa* gene expression, the three branches of QS systems had both unique and common effects on triggering phytoalexin overproduction.

To confirm the activity of phytoalexins against bacterial infection, we determined the antibacterial activity of brassilexin (ion number 36) against *P. aeruginosa* PA14 to examine its role in the *Brassica napus* defence response. It was found that brassilexin completely inhibited *P. aeruginosa* growth at a concentration of 400 µg ml^−1^, supporting its potential role seen here as an intended antibacterial defender against *P. aeruginosa* infection (Fig. 4b). When the increase in brassilexin production seen across 5 days in metabolomic profiling (Fig. S4) is paired with its demonstrated growth inhibition of *P. aeruginosa*, this strongly supports its defence role in the host–pathogen interactions seen in this study. It additionally suggests that the other identified metabolites, including the eight phytoalexins identified in this study, but particularly those that were only measurable upon the commencement of bacterial infection [including MGB (ion number 34) and brassicanal A (ion number 35)], may play a role in antibacterial defence of *Brassica napus*. Although the measured brassilexin concentration in canola exudates in the MS media (Fig. 4a) was much lower than the determined MIC against PA14, it is possible that the combination of diverse phytoalexins exuded by *Brassica napus* roots in a natural environment may be able to inhibit bacterial pathogens growth to keep potential infections at bay.

In summary, this work has identified both *P. aeruginosa* QS-associated molecules as well as host factors in the plant–bacterial interaction interface, likely playing an important role in mediating the noncanonical relationship between an important crop plant host and a unique opportunistic bacterial pathogen.

## supplementary material

10.1099/mic.0.001212 Uncited Fig. S1.

## Abbreviations

AHL: acyl-homoserine lactone
C4-HSL: *N*-butanoyl homoserine lactone
HHQ: 4-hydroxy-2-heptylquinoline
IAA: indole-3-acetic acid
LC-HRMS: liquid chromatography-high resolution MS
MGB: 4-methoxyglucobrassicin
MICA: 1-methoxy-3-indolecarboxaldehyde
3-oxo-C12-HSL: *N*-(3-oxododecanoyl) homoserine lactone
PCA: phenazine-1-carboxylic acid
PLS-DA: partial least squares-discriminant analysis
PQS: 2-heptyl-3-hydroxy-4-quinolone
QS: quorum sensing
