# Supplementary material for: Production of quorum sensing-related metabolites and phytoalexins during Pseudomonas aeruginosa–Brassica napus interaction
Source: Microbiology (Reading). 2022 Aug 18;168(8):001212. doi: 10.1099/mic.0.001212 (PMC11449044; doi:10.1099/mic.0.001212)
Supplement: Uncited Fig. S1. [file mic-168-01212-s001.pdf]

Supplemental information

Figure S1: ***P. aeruginosa*** **PA14 growth in the absence and presence of canola over a 5-day interaction.** Bacterial counting of the initial inoculum and bacterial controls (BC) in MS media without plants and bacteria grown in the presence of canola in MS media (Infected) on Days 1, 3 and 5. Bacterial counting was calculated as Log_{10} CFU/mL. Data are the means of three biological replicates with the error bars representing standard errors.

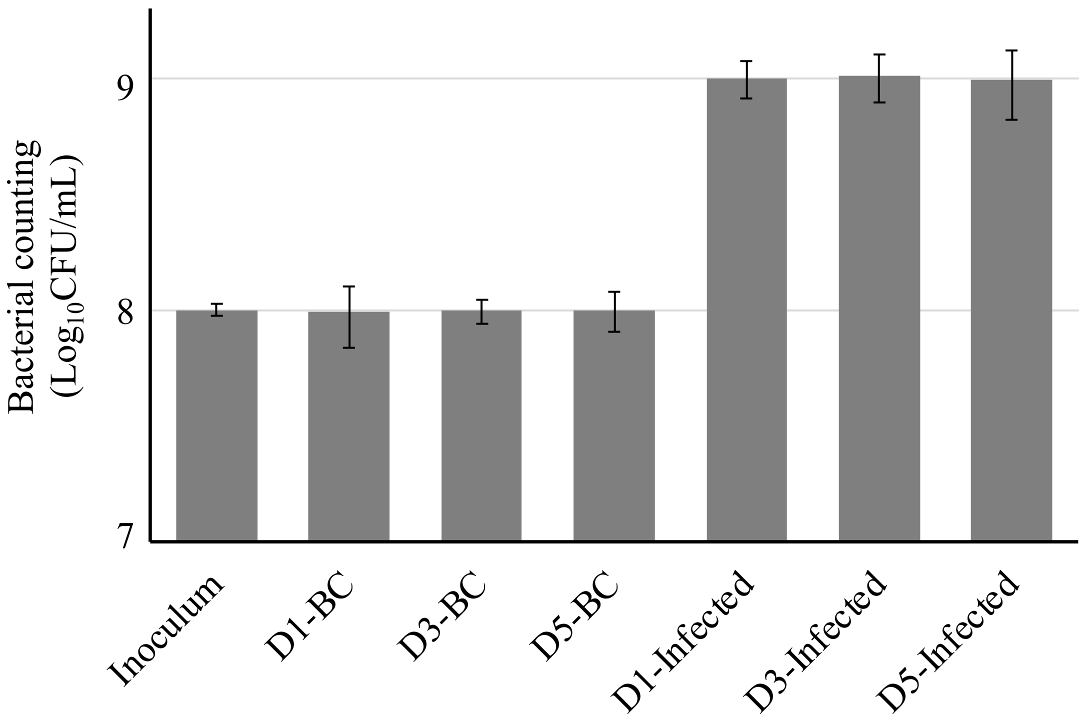

Figure S2: Confirmation (LC-HRMS and MS/MS) of metabolites C4-HSL, 3-oxo-C12-HSL, PQS, pyocyanin, HHQ, rhamnolipids RL1 and RL2 using reference standards. Confirmation of C4-HSL, 3-oxo-C12-HSL, PQS, pyocyanin, HHQ, rhamnolipids RL1 and RL2 with commercially available standards for retention time (LC-HRMS) and MS/MS fragmentation patterns was carried out by using a D5-Infected sample as a representative. The extracted ion chromatograms (LC-HRMS, A) of the targeted metabolites and the MS/MS fragmentation patterns of the precursor ions (MS/MS, B) were compared with the reference standards. The major fragment is shown as circled with proposed fragmentation schemes shown as inserts. In the infected samples when ion abundance is low in the chromatogram due to other adjacent ions with high abundance, an arrow indicates the location of the target ion.

Figure S2-1: C4-HSL

A

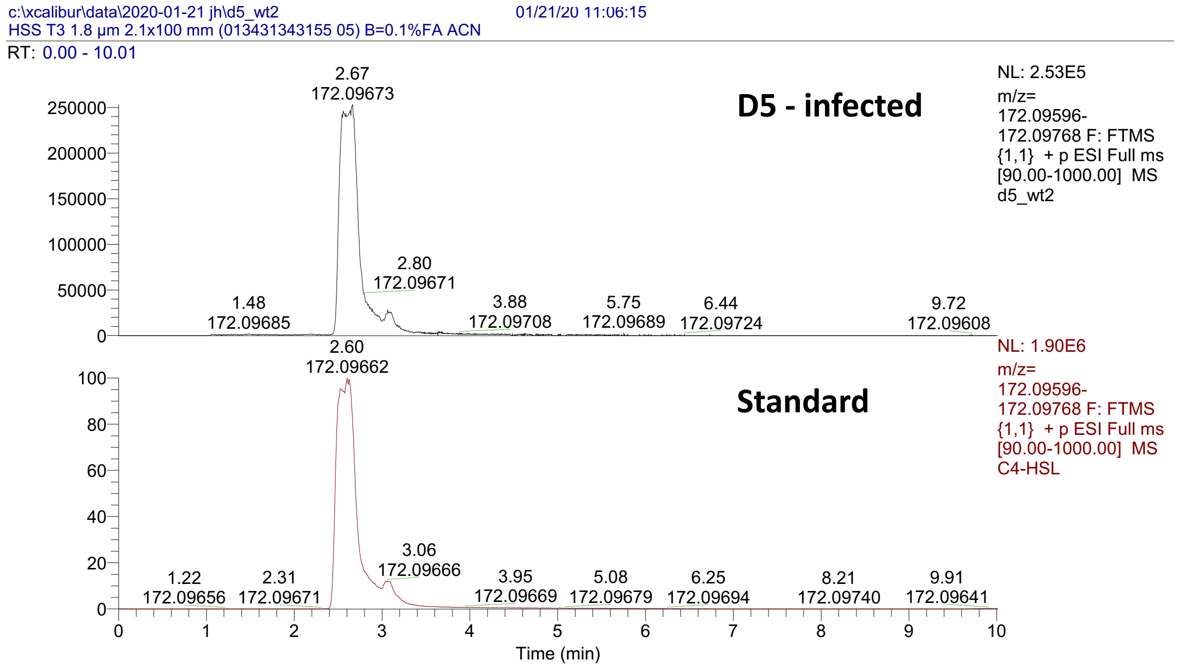

B

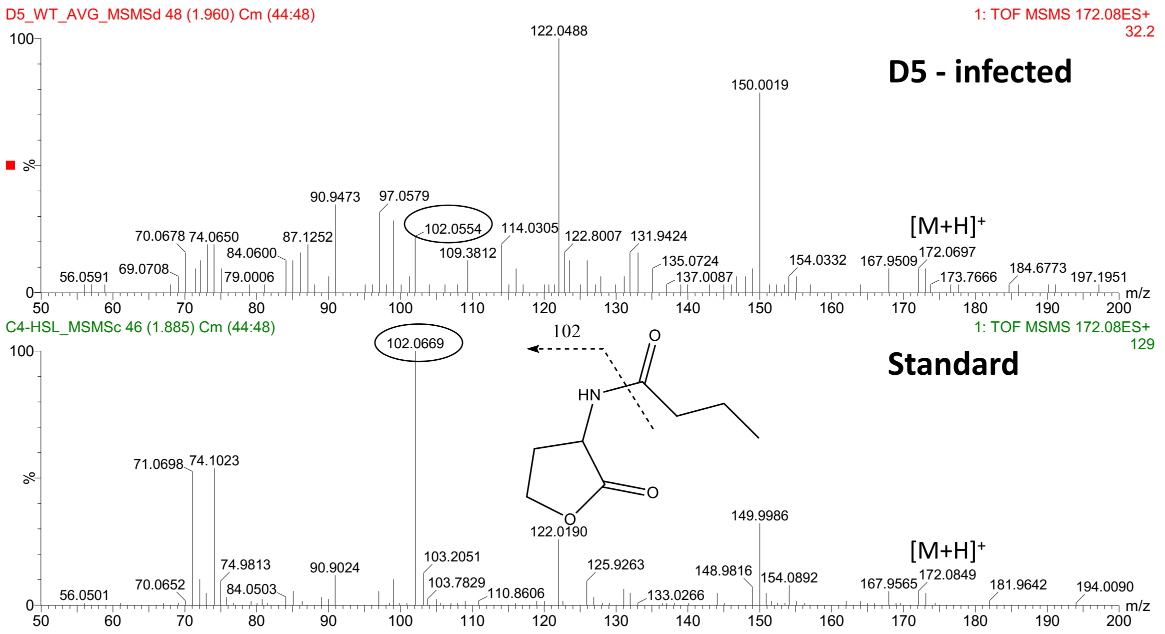

Figure S2-2: 3-oxo-C12-HSL

A

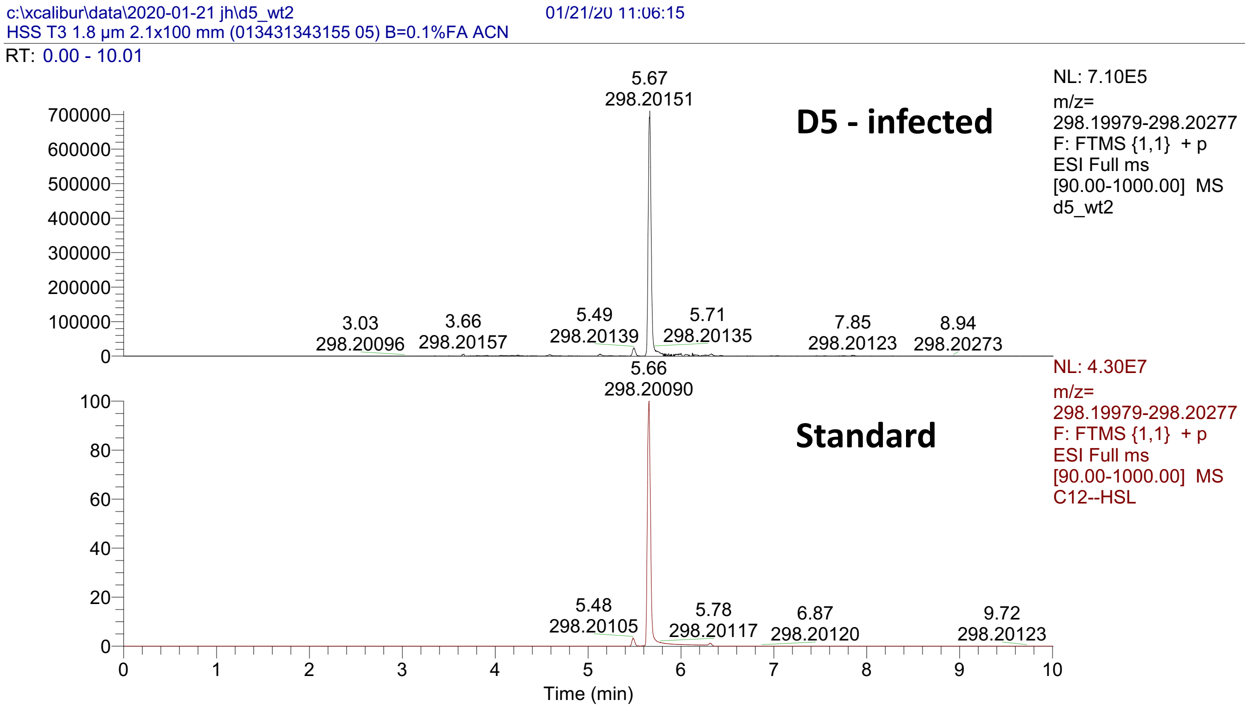

B

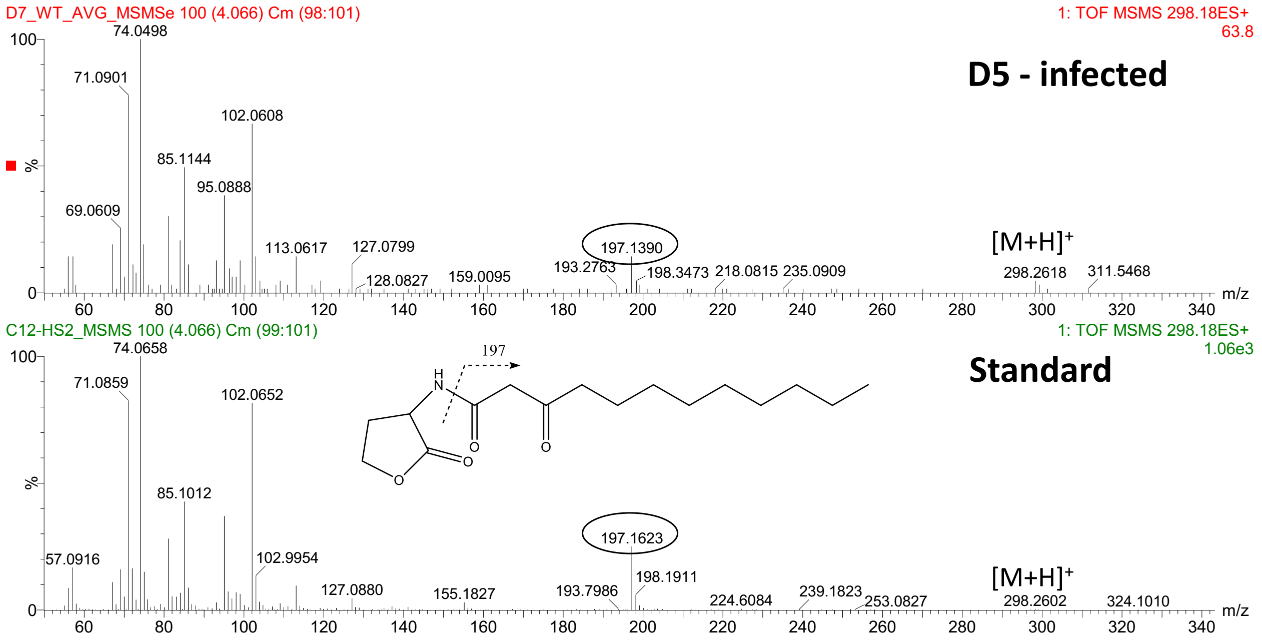

Figure S2-3: PQS

A

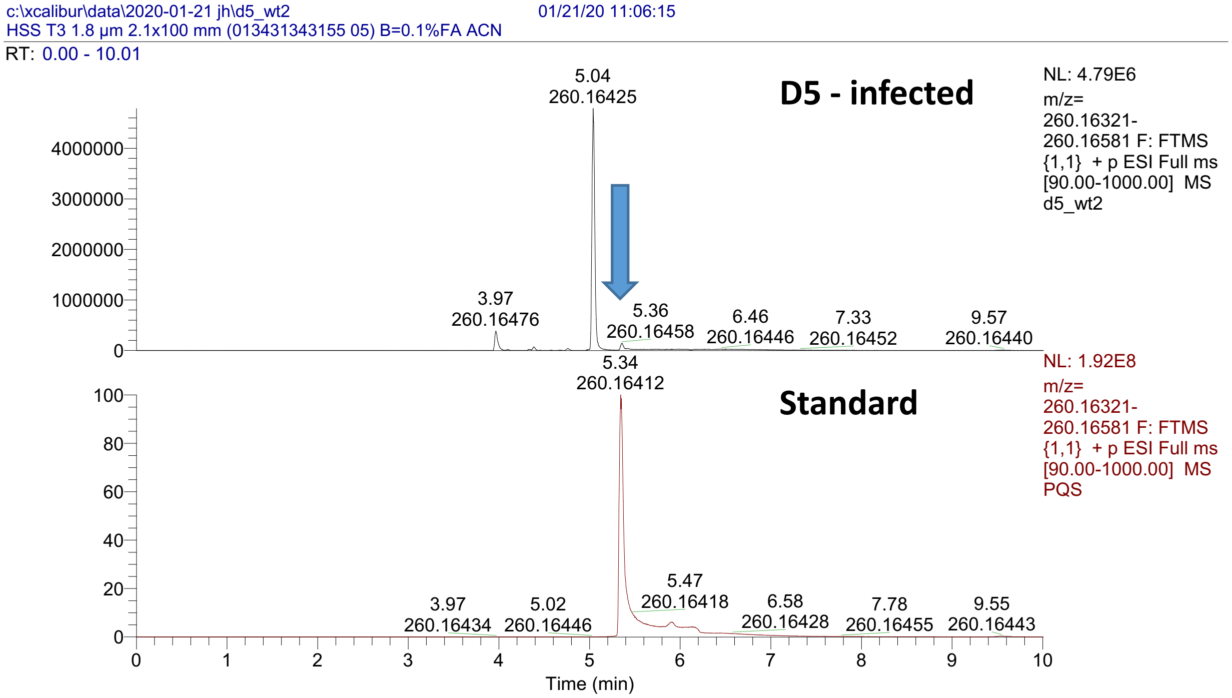

B

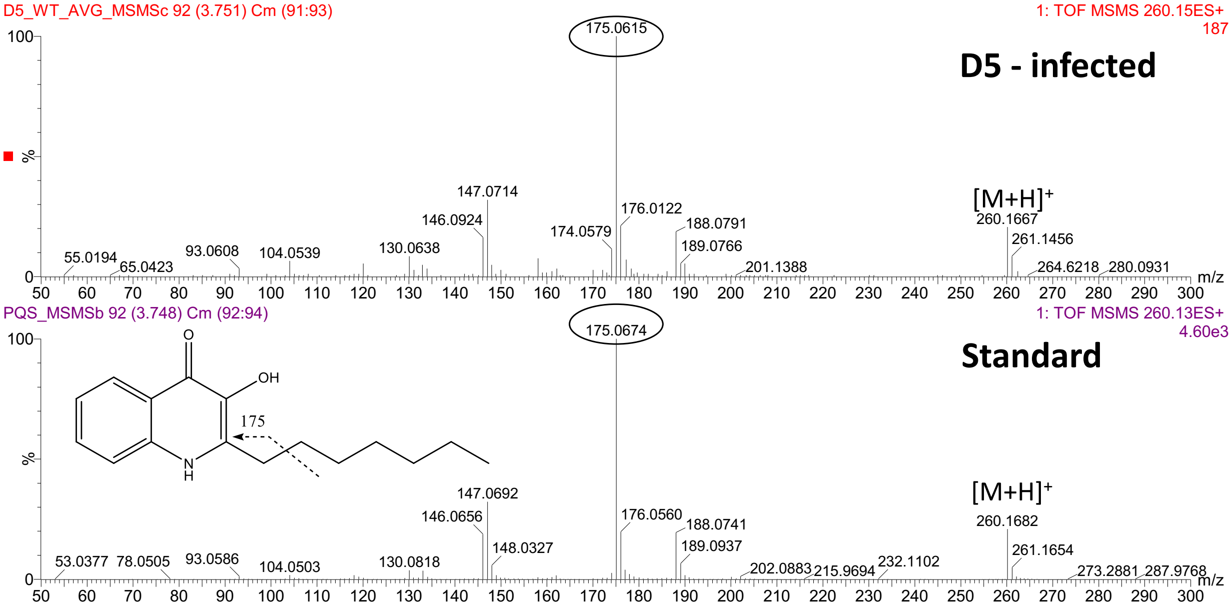

Figure S2-4: Pyocyanin

A

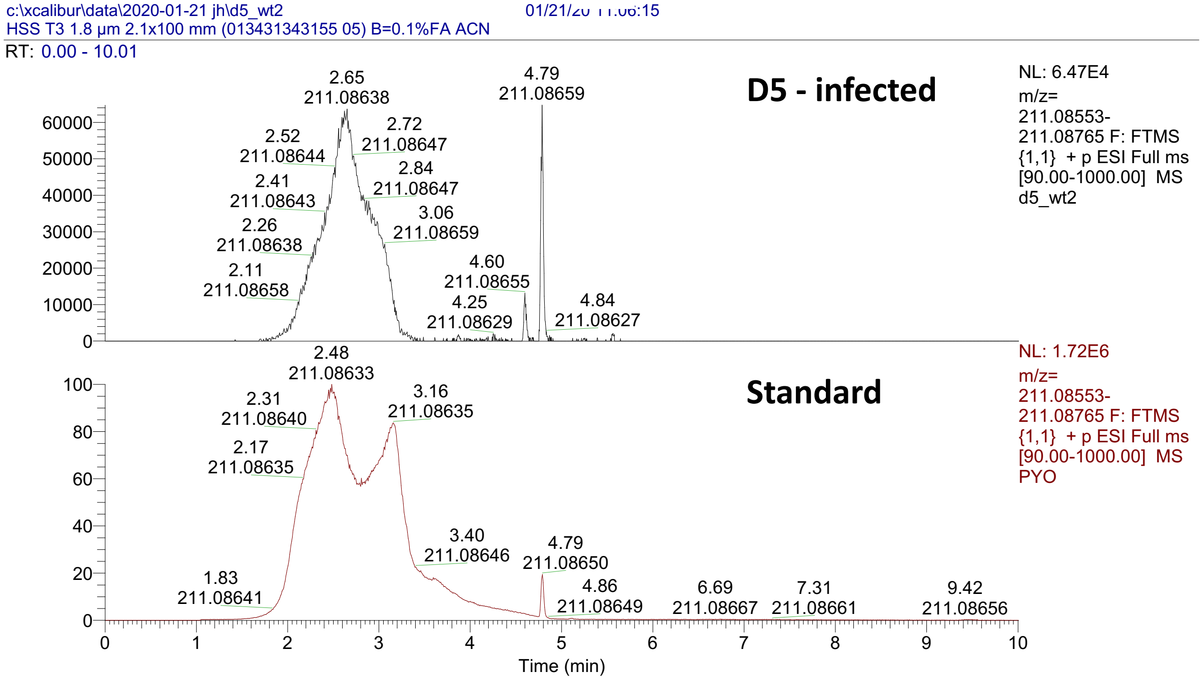

B

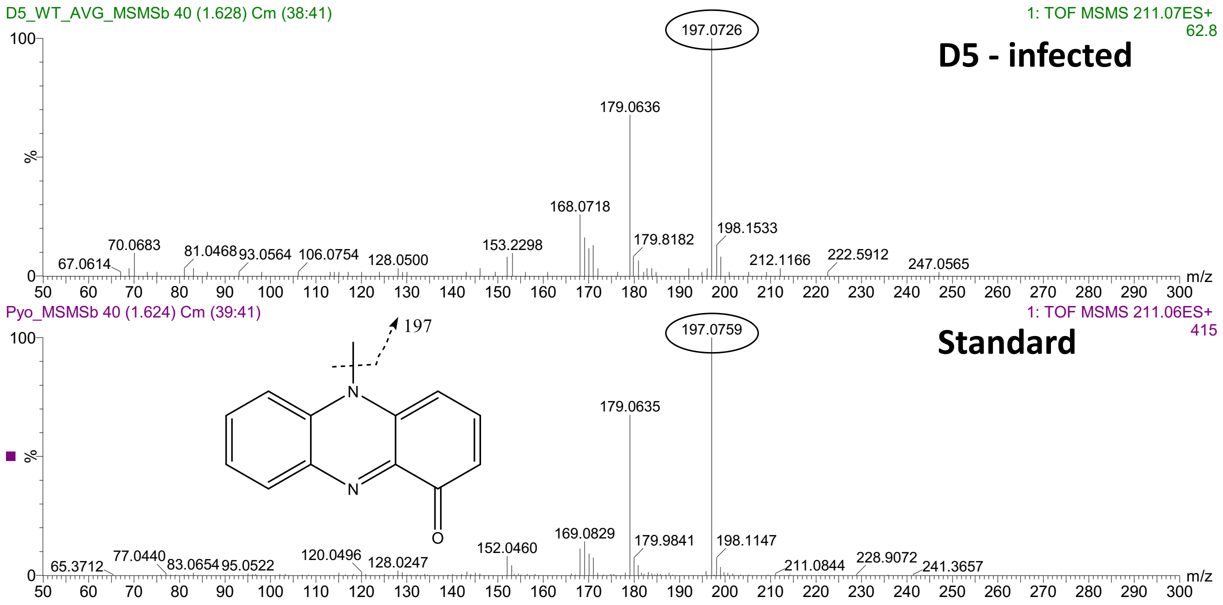

Figure S2-5: HHQ

A

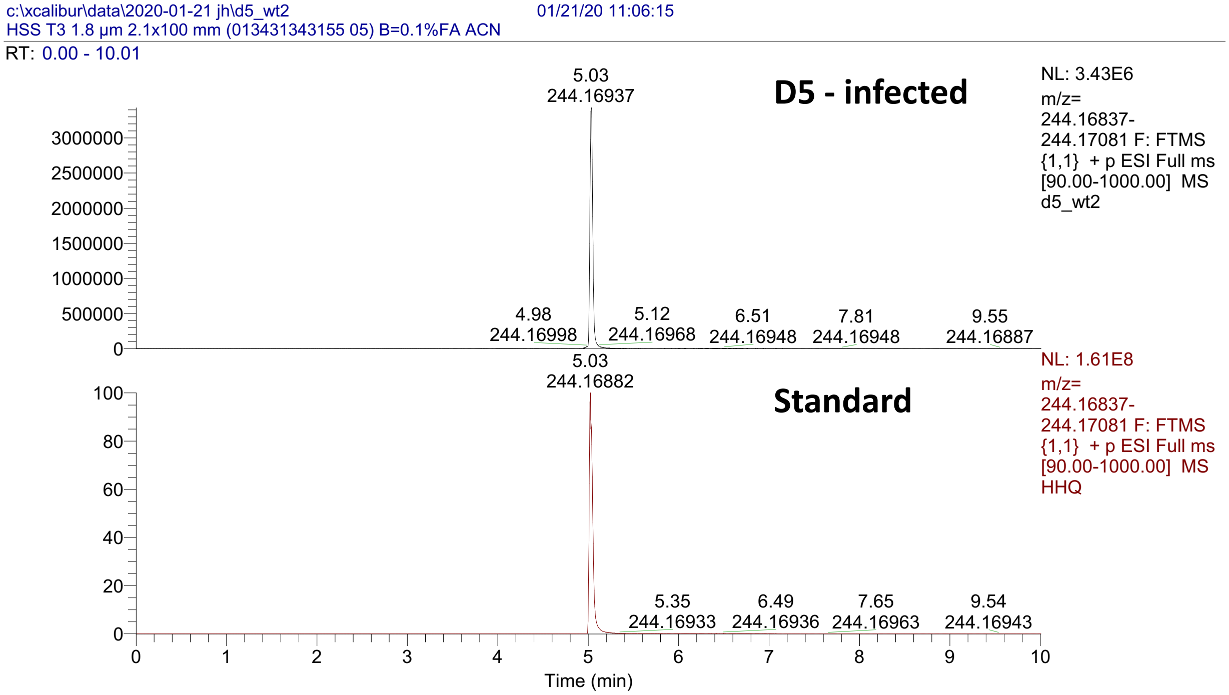

B

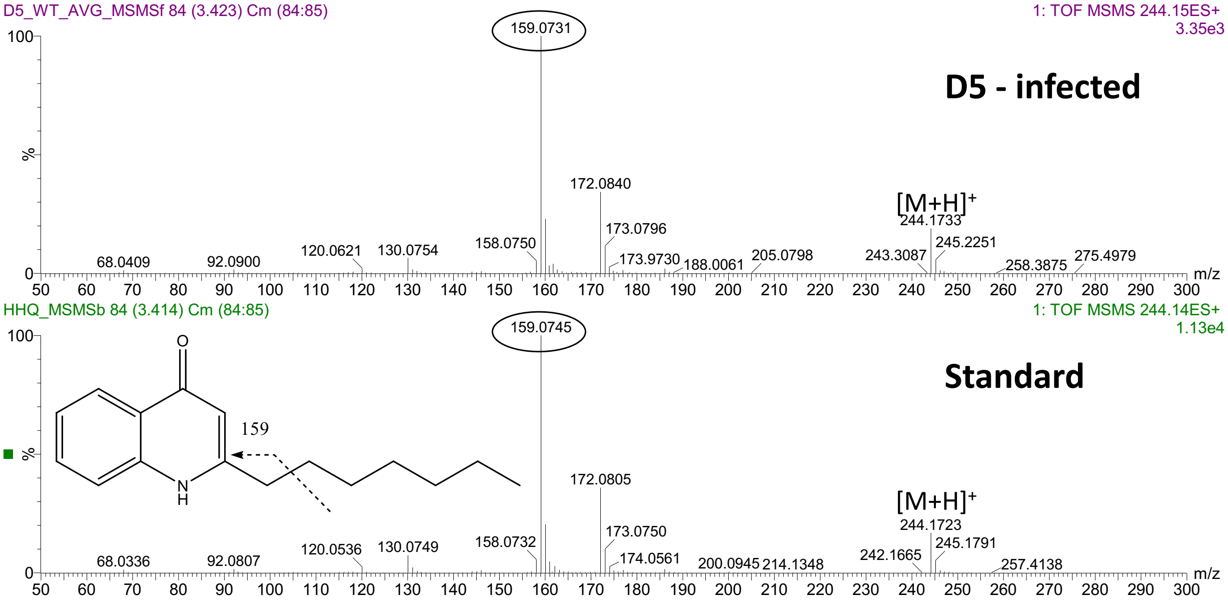

Figure S2-6: Rhamnolipids RL1

A

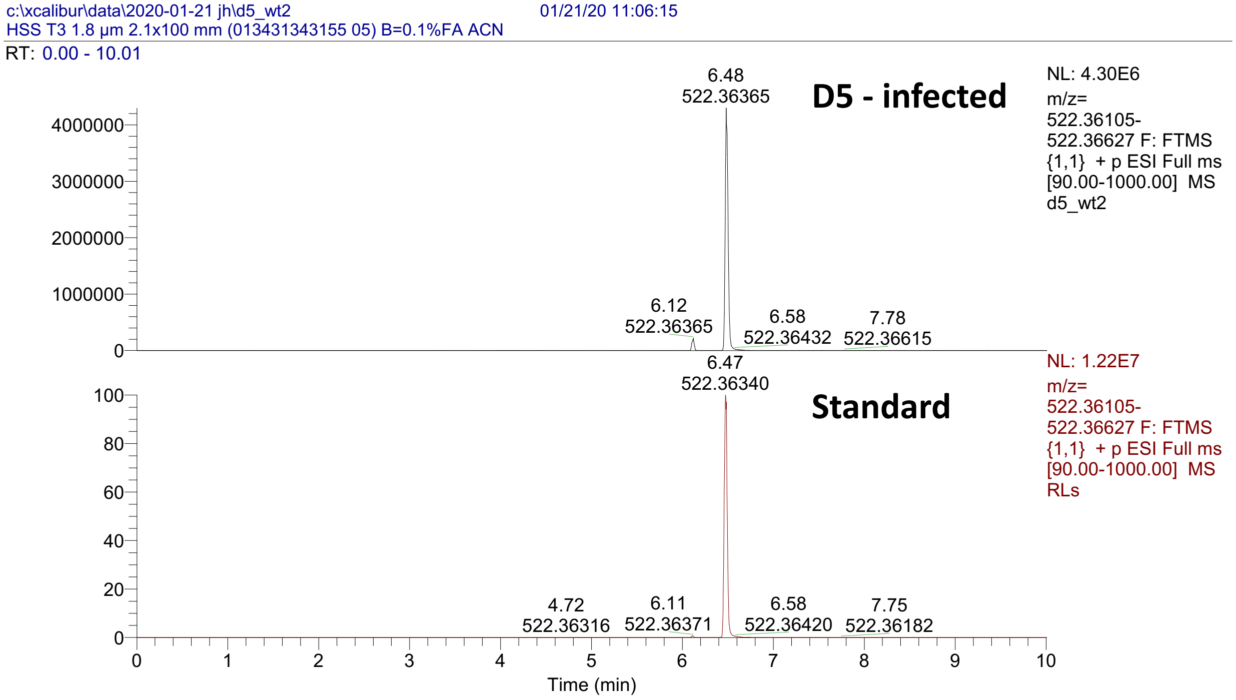

B

MS/MS data not available

Figure S2-7: Rhamnolipids RL2

A

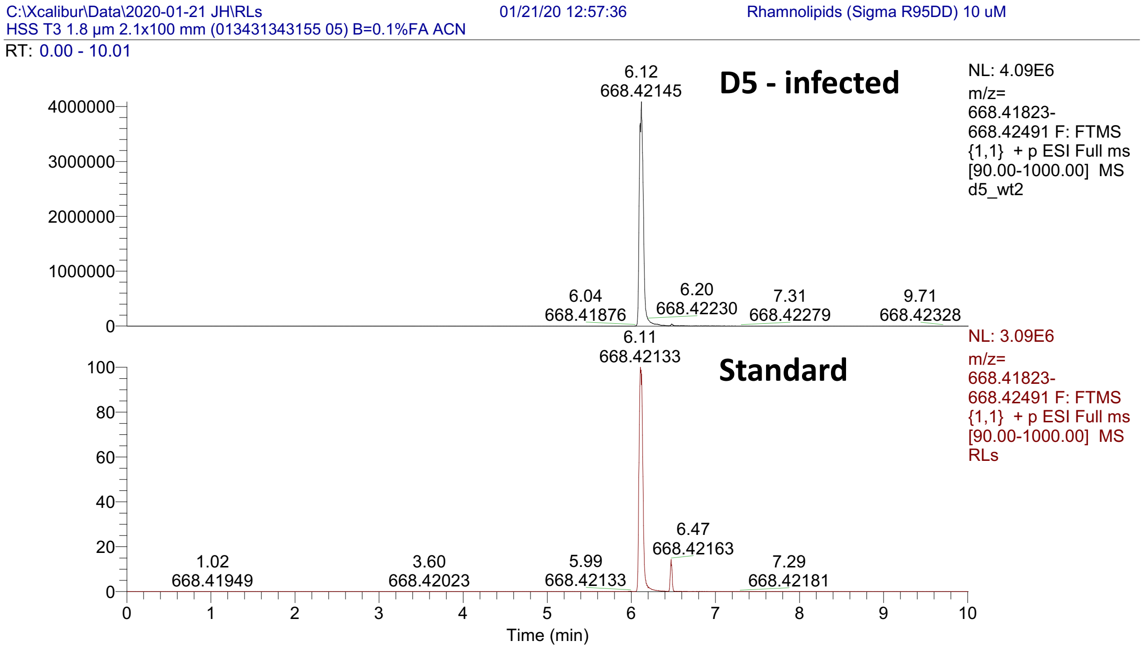

B

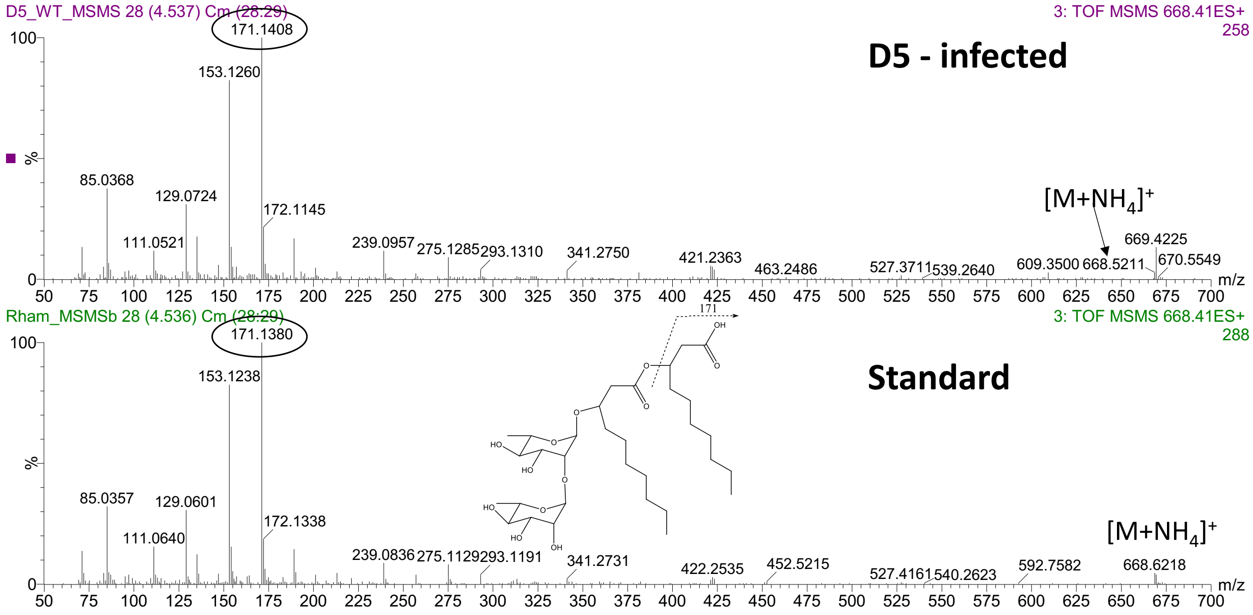

Figure S3: Changes in levels of *Pseudomonas aeruginosa* metabolites tentatively identified. Box plots on levels of *Pseudomonas aeruginosa* metabolites in growth media of canola seedling with *P. aeruginosa* infection. Y axis displays the peak area of each metabolite normalized using reserpine as an internal standard. Center lines show the medians; box limits indicate the 25th and 75th percentiles as determined by R software; whiskers extend 1.5 times the interquartile range from the 25th and 75th percentiles; and outliers are represented by dots. n = 4, which include 4 biological replicates, each with duplicated LC-MS analysis. D1, D3, D5 represent the samples on day 1, day 3, and day 5 post *P. aeruginosa* infection, respectively. BC, bacterial control. Infected, canola with *P. aeruginosa* infection. Metabolite levels were assessed by student's t test. * compared with BC; § compared with D1-infected sample. * or § $P < 0.05$; ** $P < 0.01$; *** $P < 0.0001$; **** or §§§§ $P < 0.00001$. ND, not detectable in the D1-infected samples.

Figure S3-1. Quinolones - 4-hydroxy-2-heptylquinoline (HHQ) (Ion 12, variable ID 280)

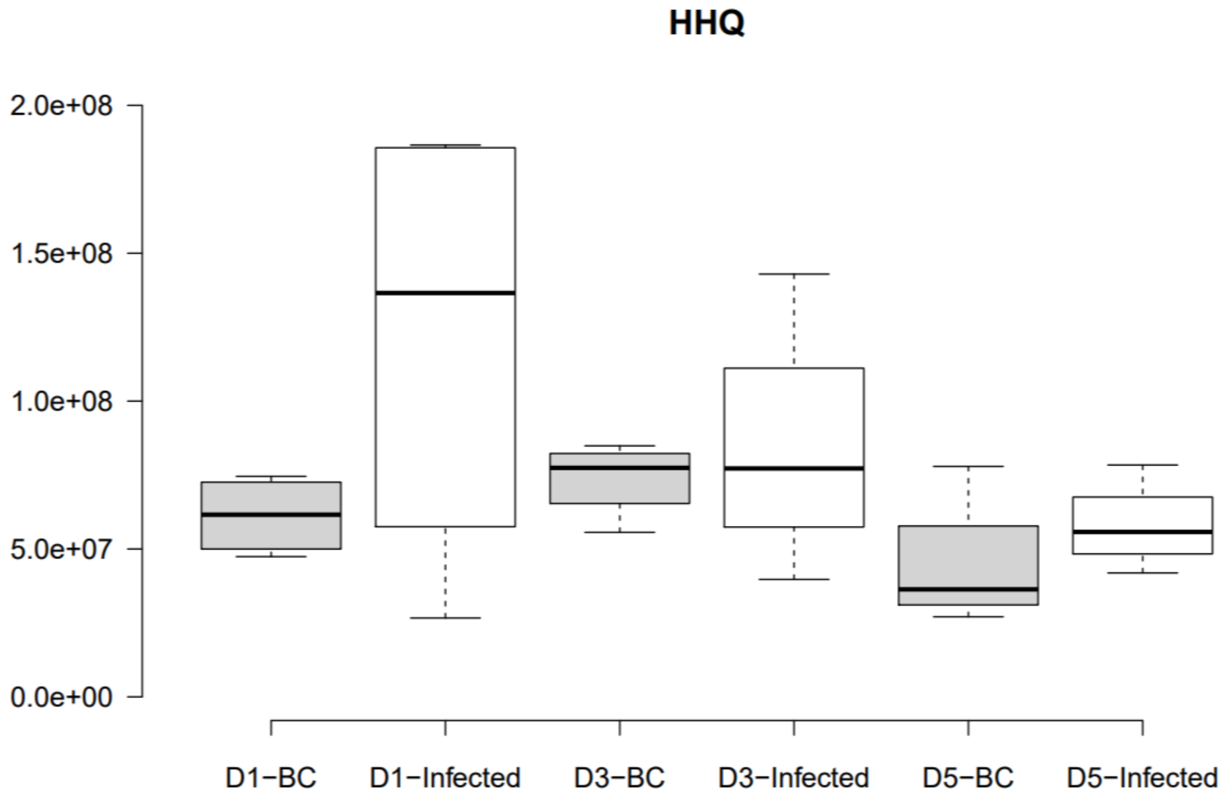

Figure S3-2. Quinolones – 2-Heptyl-4-hydroxyquinoline-N-oxide (HQNO) (Ion 13, variable ID 295)

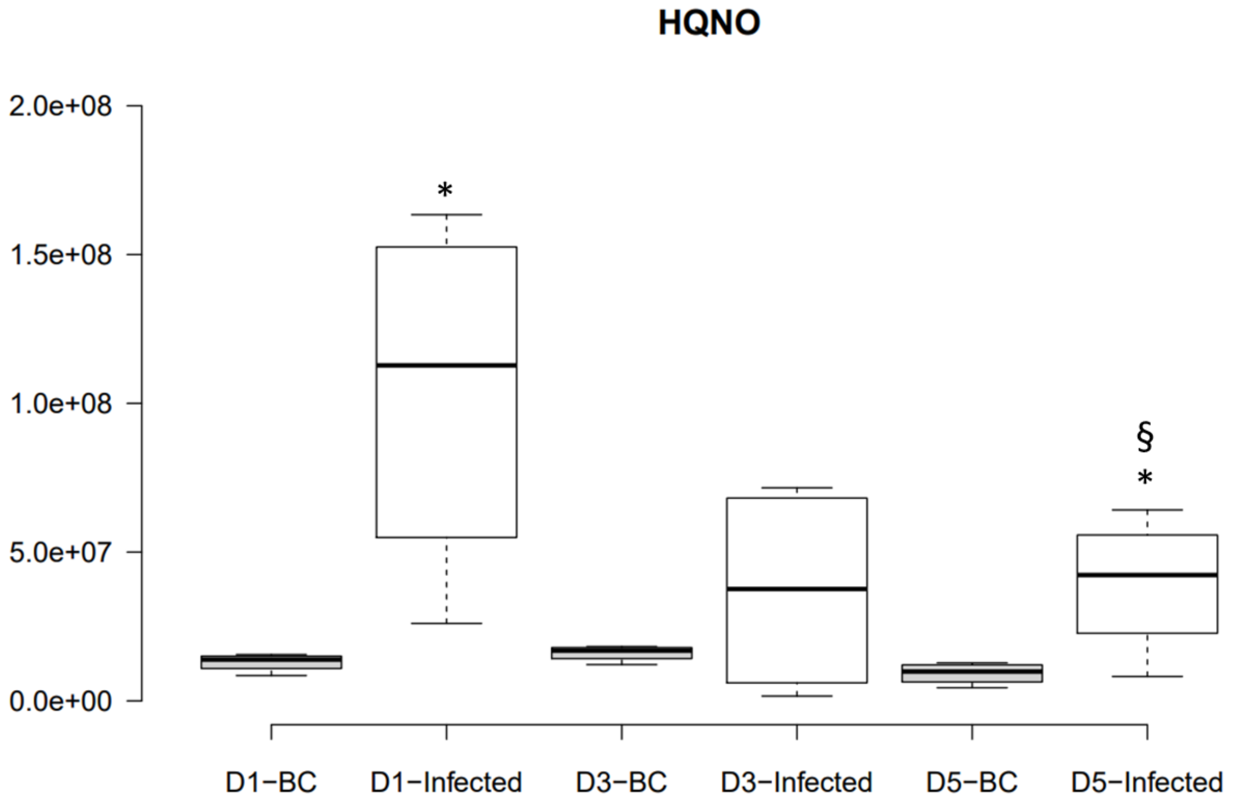

Figure S3-3. Quinolones – 4-Hydroxy-2-nonenylquinoline or isomer (Ion 16, variable ID 281)

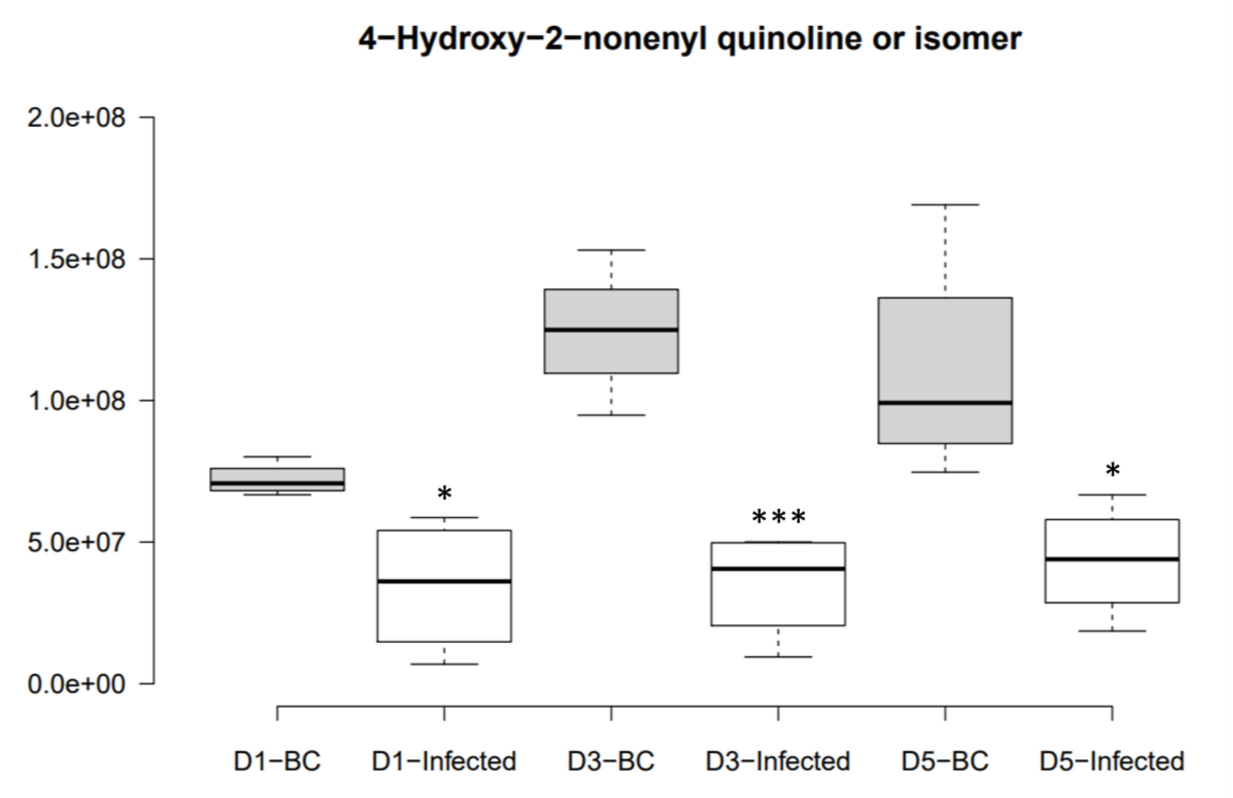

Figure S3-4. Quinolones – 4-Hydroxy-2-nonenylquinoline or isomer (Ion 17, variable ID 277)

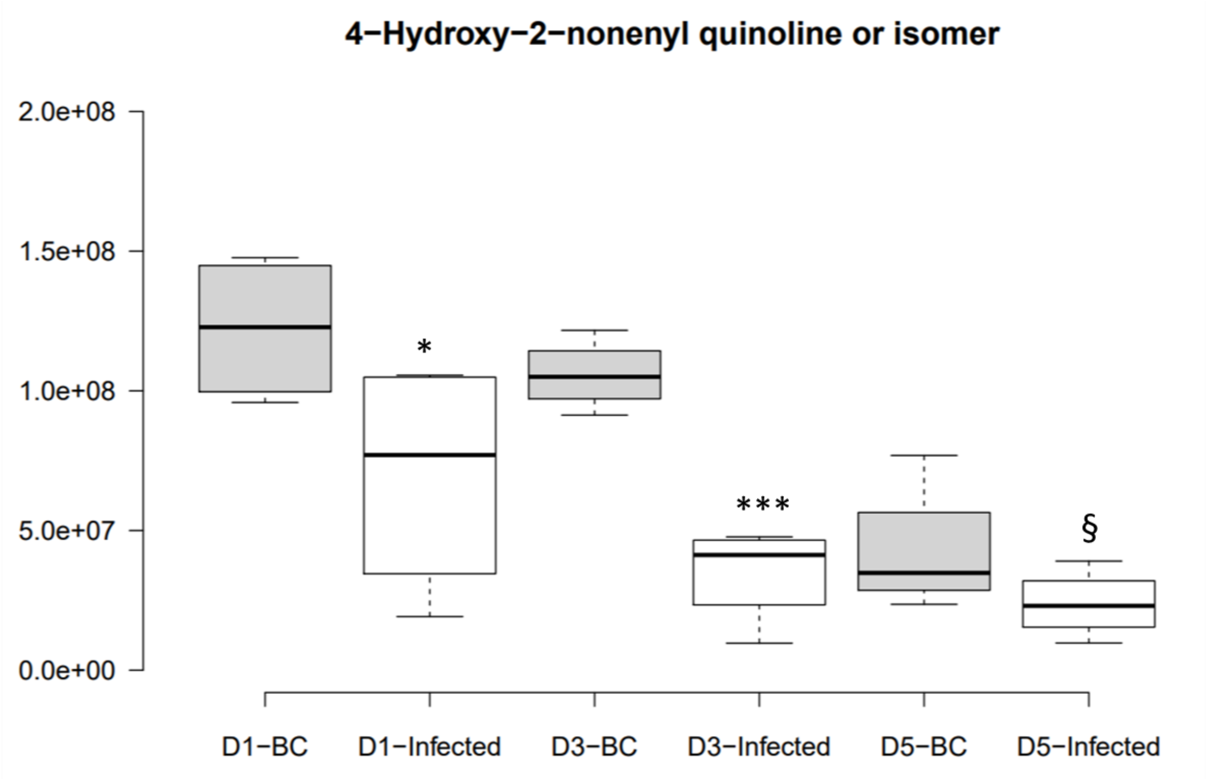

Figure S3-5. Quinolones – 4-Hydroxy-2-nonylquinoline (HNQ) (Ion 18, variable ID 276)

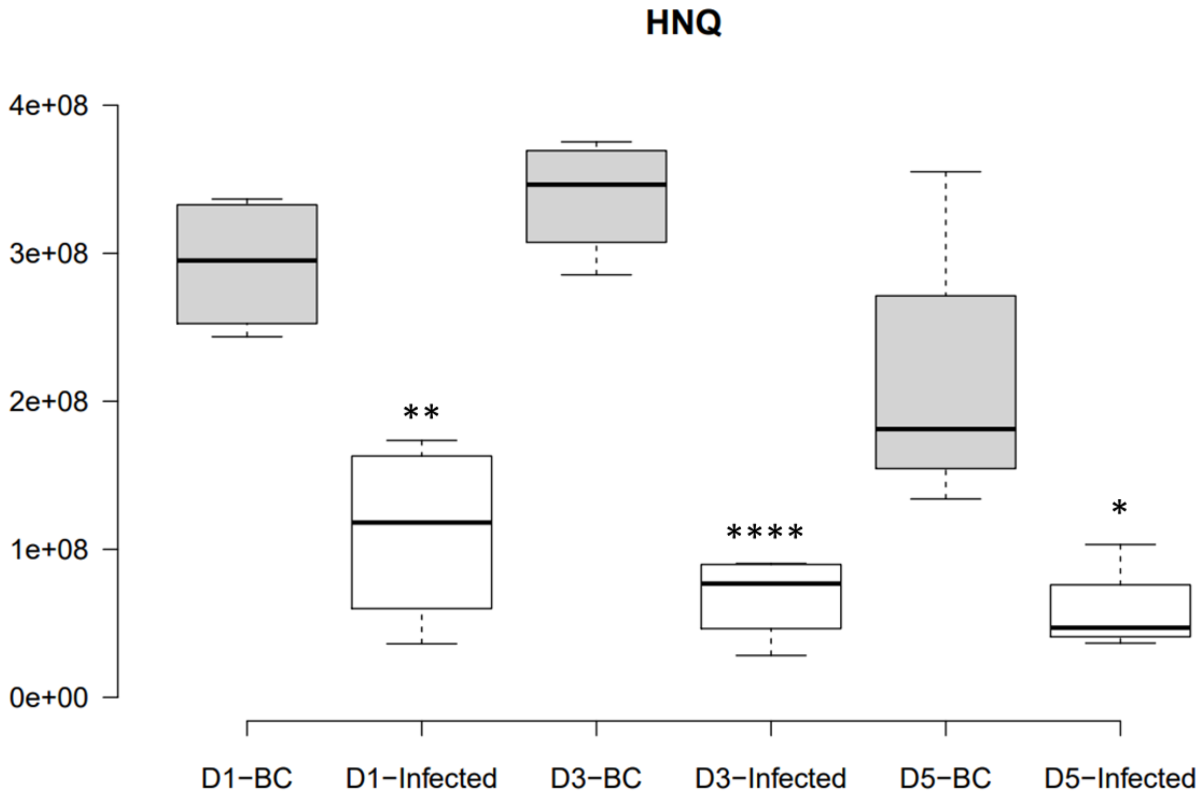

Figure S3-6. Phenazines – Phenazine-1-carboxamide (PCN) (Ion 5, variable ID 655)

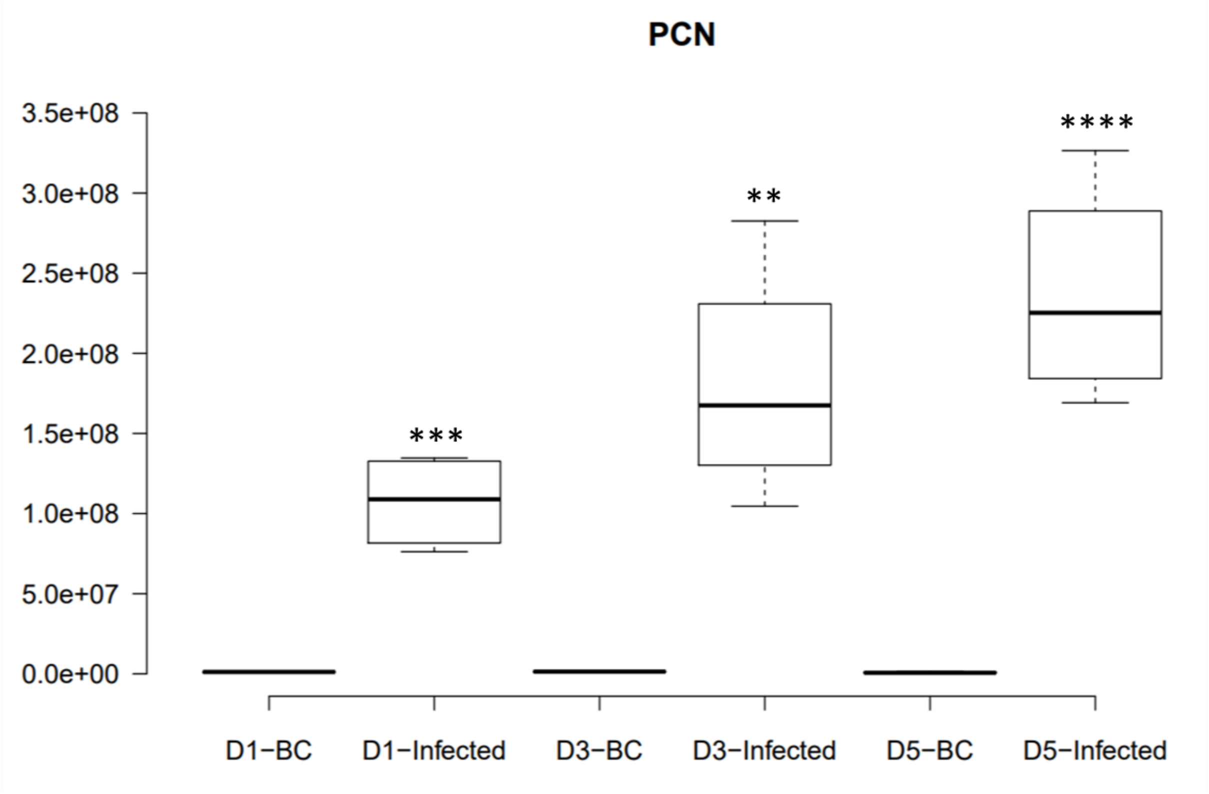

Figure S3-7. Phenazines – Phenazine-1-carboxylic acid (PCA) (Ion 10, variable ID 354)

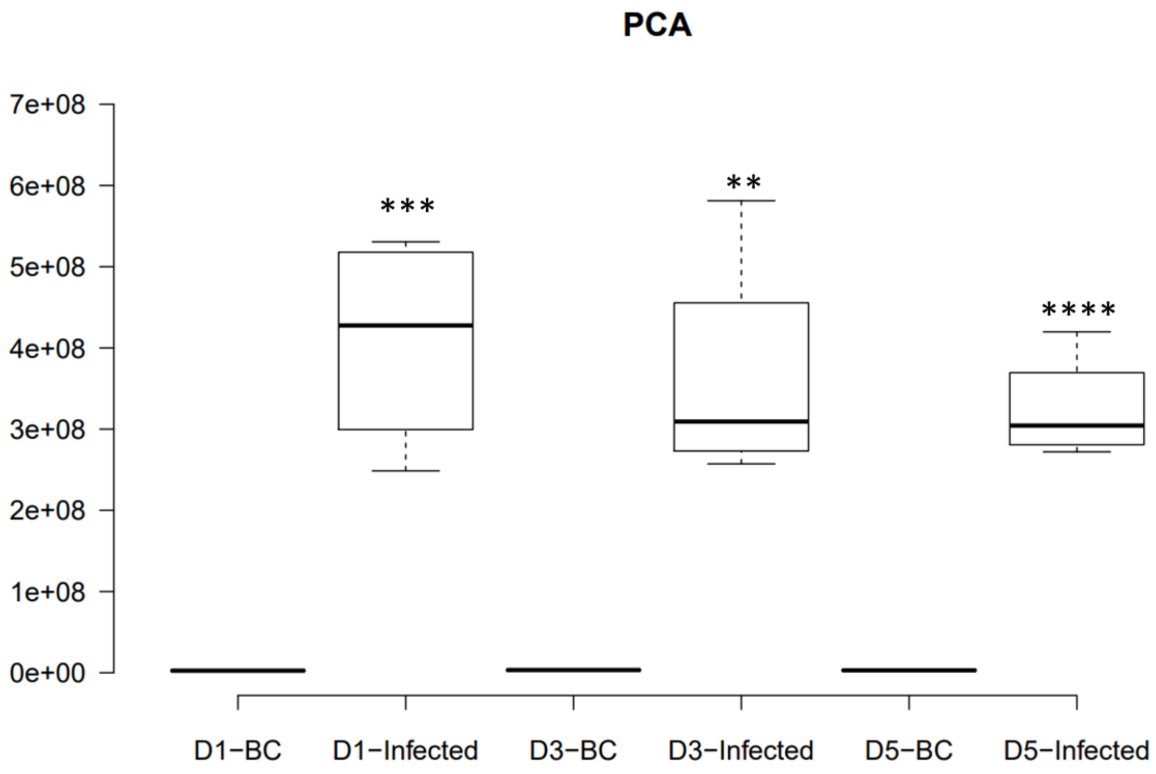

Figure S3-8. Phenazines – 5-Methyl-phenazine-1-carboxylic acid (Ion 14, variable ID 1808)

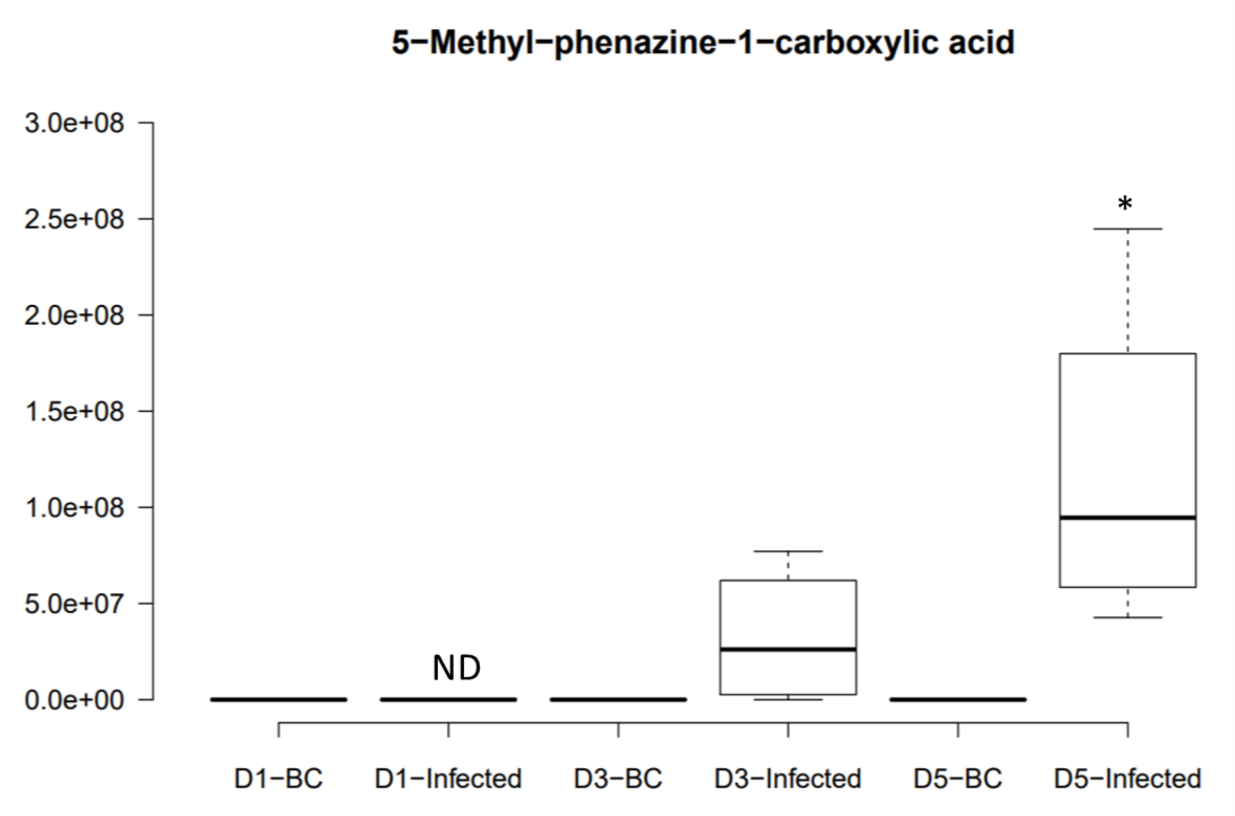

Figure S3-9. Phenazines – 1-Carbomethoxyphenazine (Ion 19, variable ID 683)

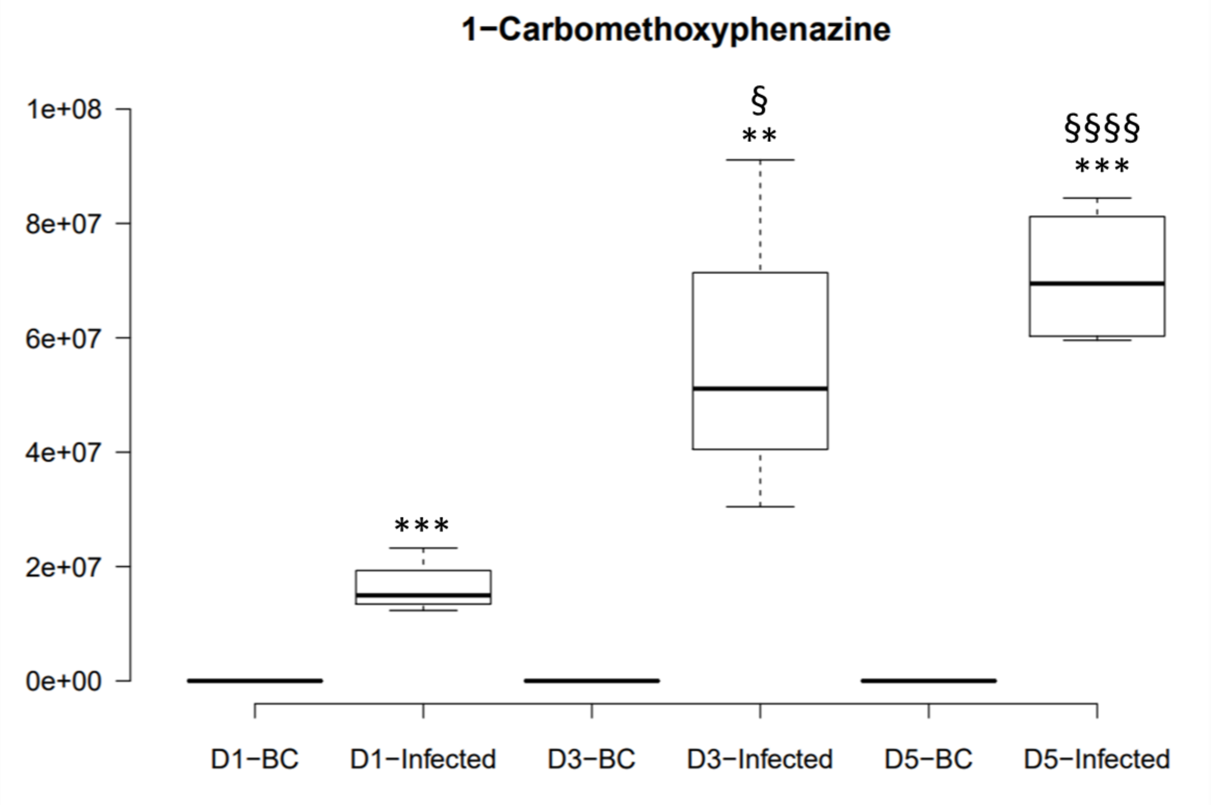

Figure S3-10. Rhamnolipids – RL MW676 (Ion 28, variable ID 699)

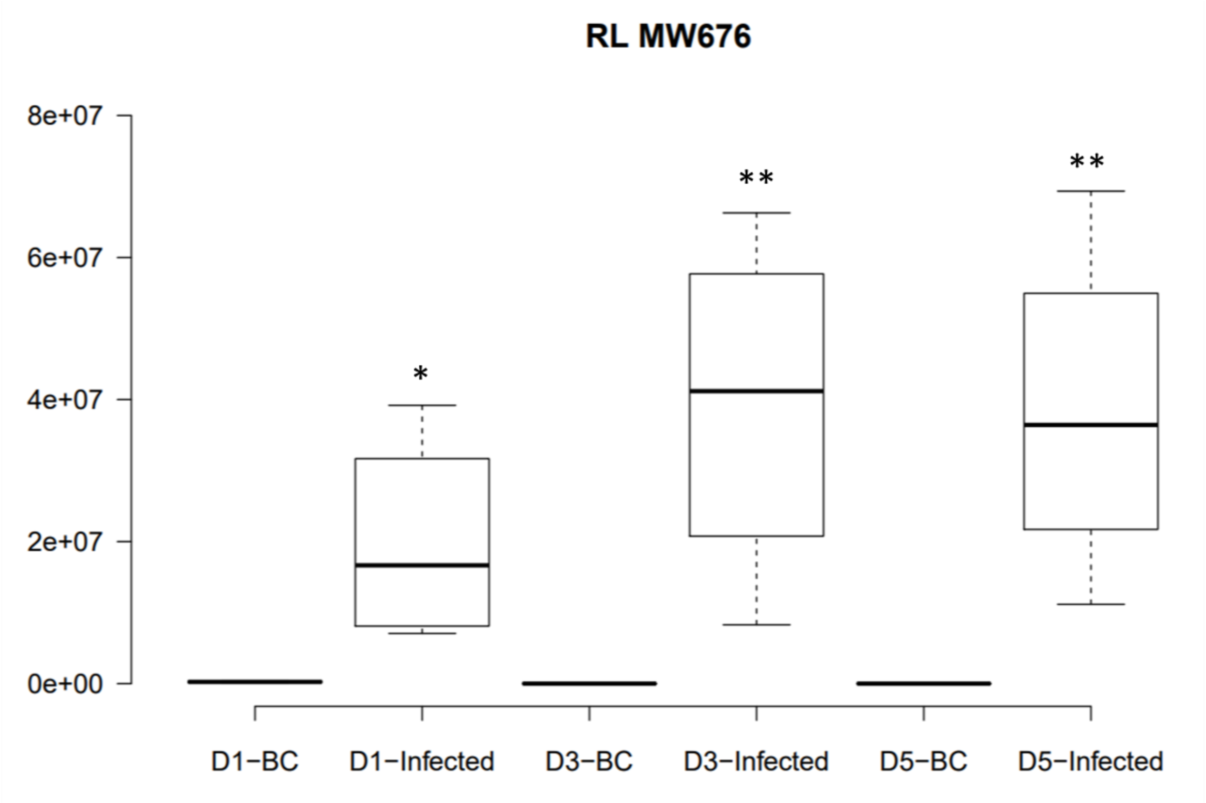

Figure S3-11. Rhamnolipids – RL MW678 (Ion 32, variable ID 697)

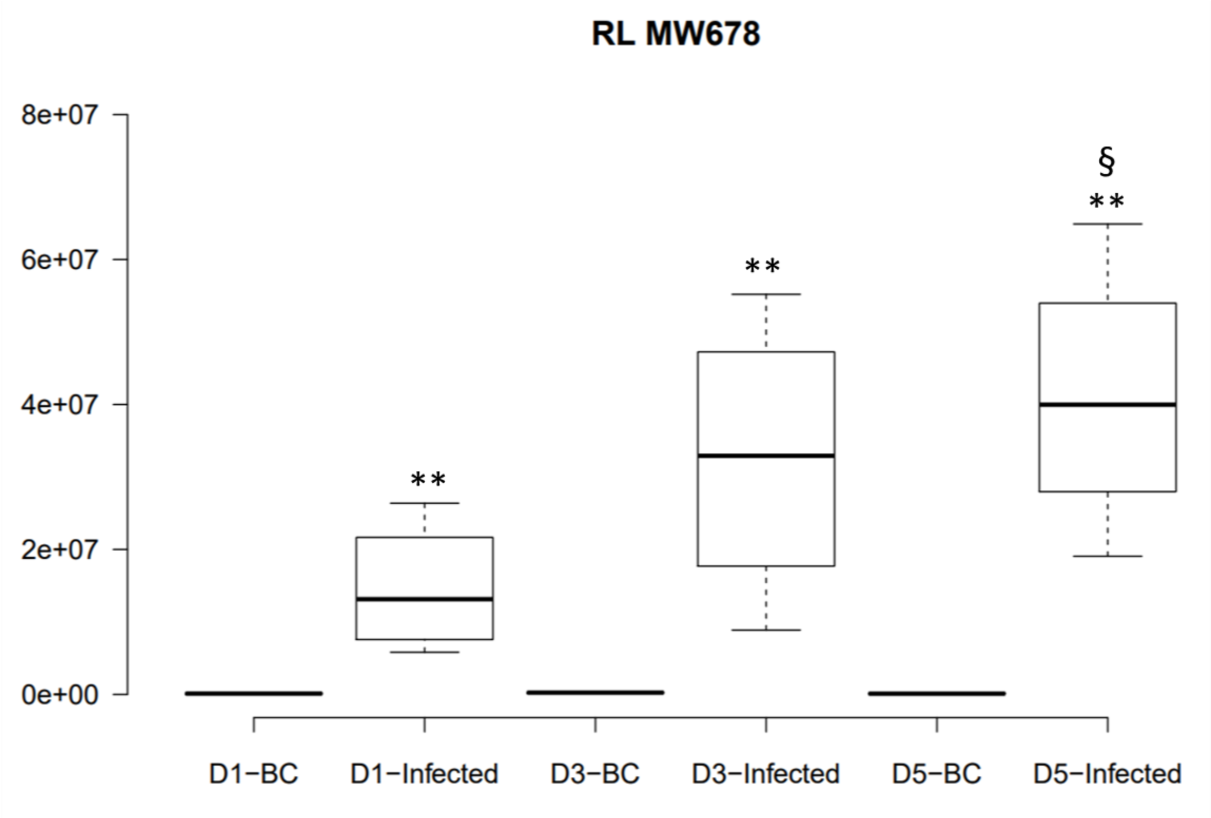

Figure S3-12. Other - Pyochelin (Ion 15, variable ID 278)

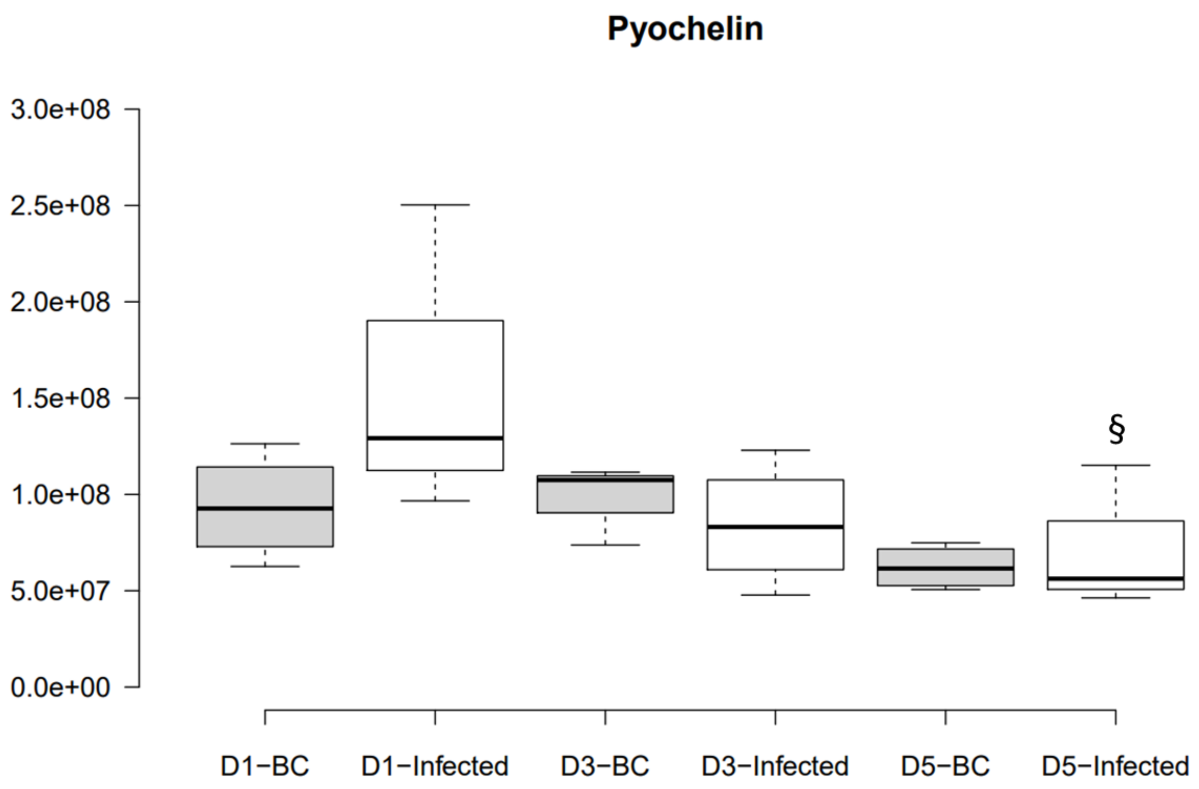

Figure S4: Changes of canola root exuded metabolite levels. Box plots showing the levels of canola root exuded metabolites in growth media of canola seedling with *P. aeruginosa* infection. Y axis displays the peak area of each target metabolite normalized using reserpine as an internal standard. Center lines show the medians; box limits indicate the 25th and 75th percentiles as determined by R software; whiskers extend 1.5 times the interquartile range from the 25th and 75th percentiles; and outliers are represented by dots. n = 4, which include 4 biological replicates, each with duplicated LC-MS analysis. D1, D3, D5 represent the samples on day 1, day 3, and day 5 post *P. aeruginosa* infection, respectively. BC, bacterial control. Infected, canola with *P. aeruginosa* infection. PC, canola plant control. Metabolite levels were assessed by student's t test. * compared with PC; § compared with D1-infected sample. * or § $P < 0.05$; ** or §§ $P < 0.01$; *** or §§§ < 0.0001; **** or §§§§ $P < 0.00001$. ND, not detectable in the D1-infected samples.

Figure S4-1: Indole-3-acetaldehyde (Ion 3, variable ID 662)*

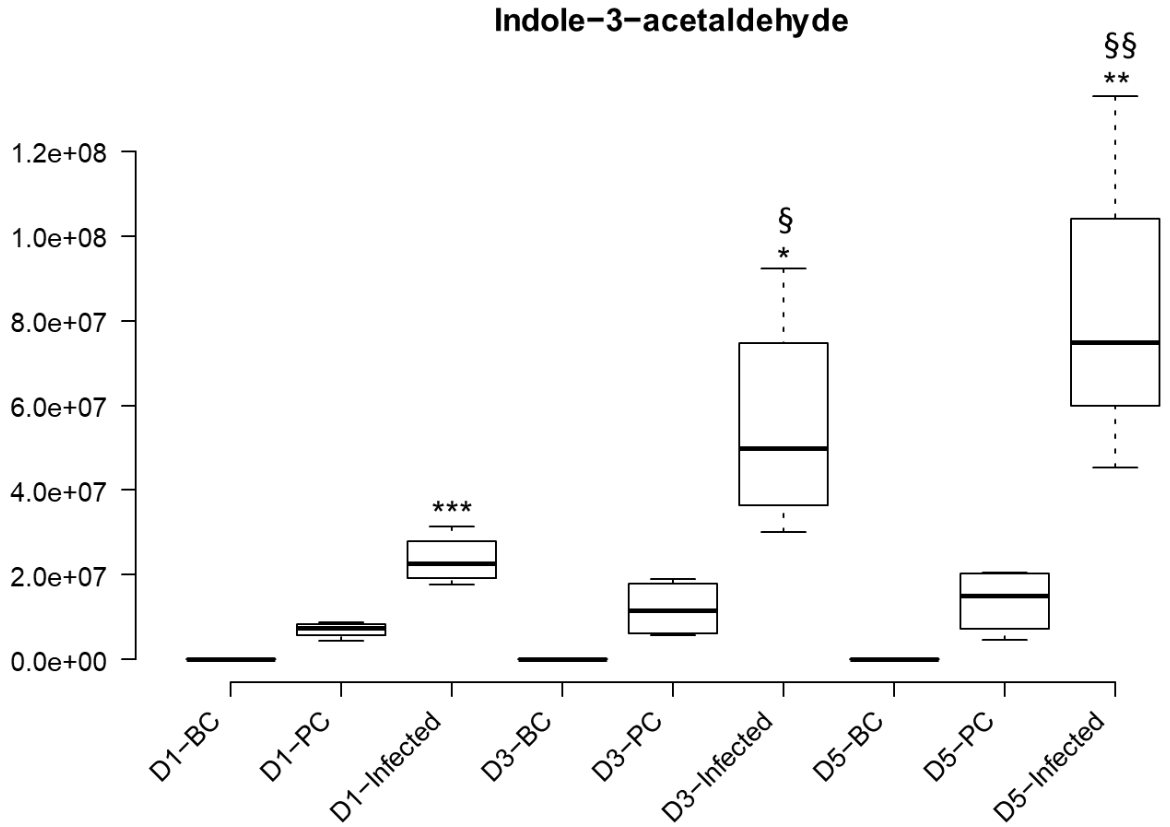

*This metabolite may also be from *P. aeruginosa*

Figure S4-2: Cyclic spermidine conjugate (Ion 4, variable ID 795)

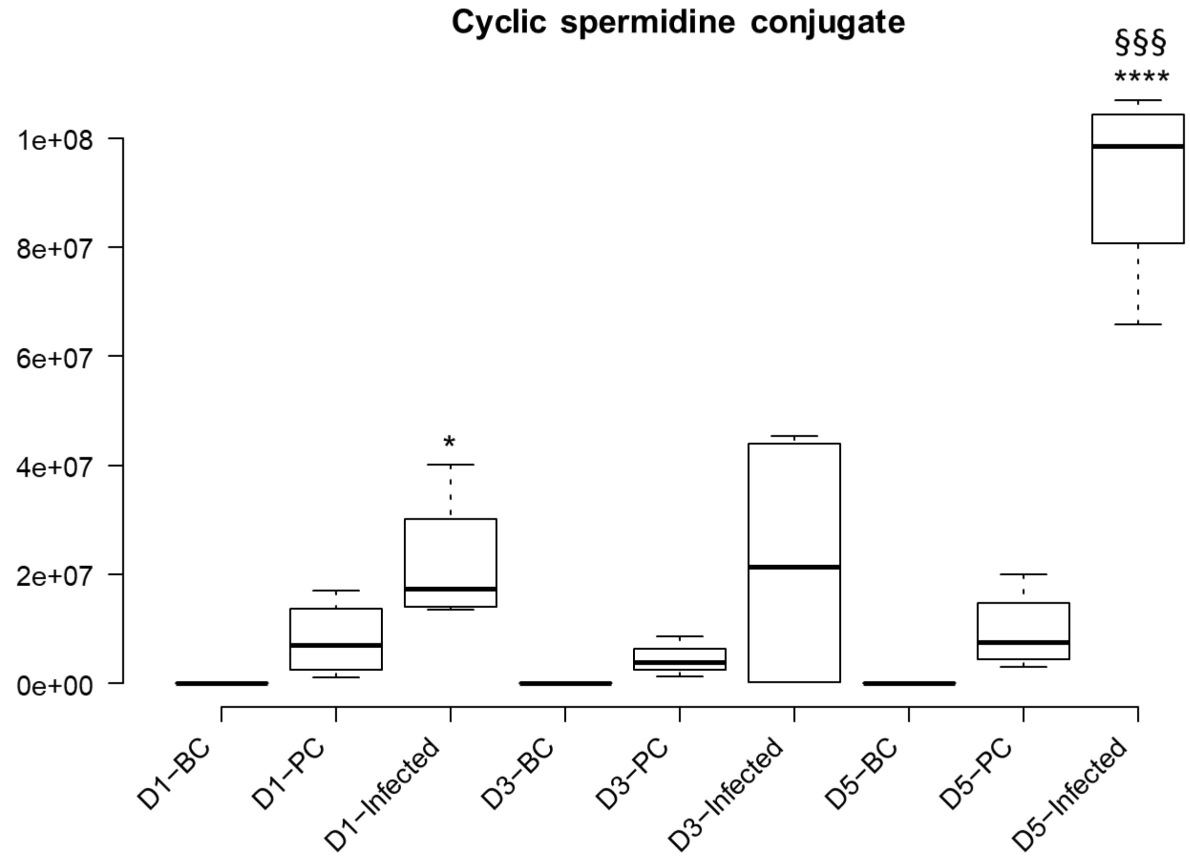

Figure S4-3: 4-Methoxyglucobrassicin (MGB) (Ion 34)

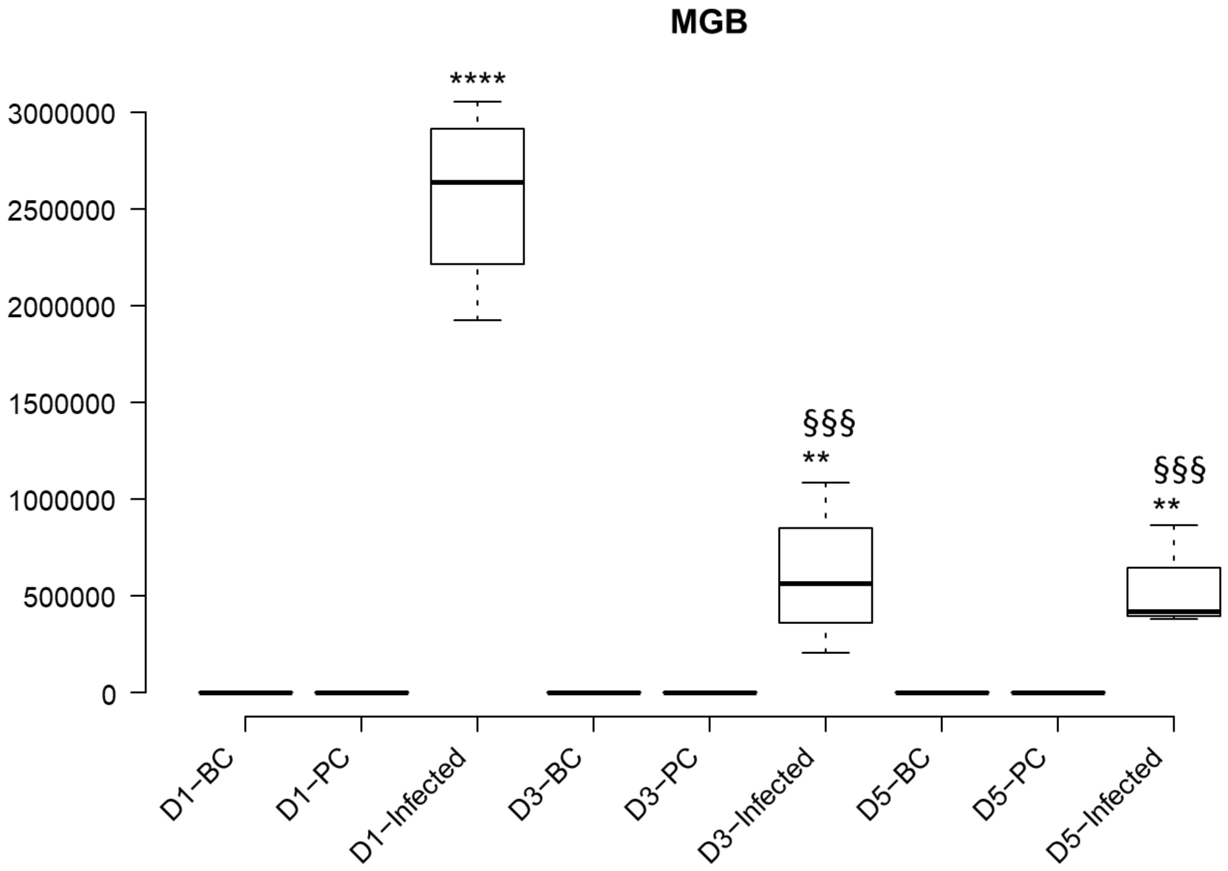

Figure S4-4: Brassicanal A (Ion 35)

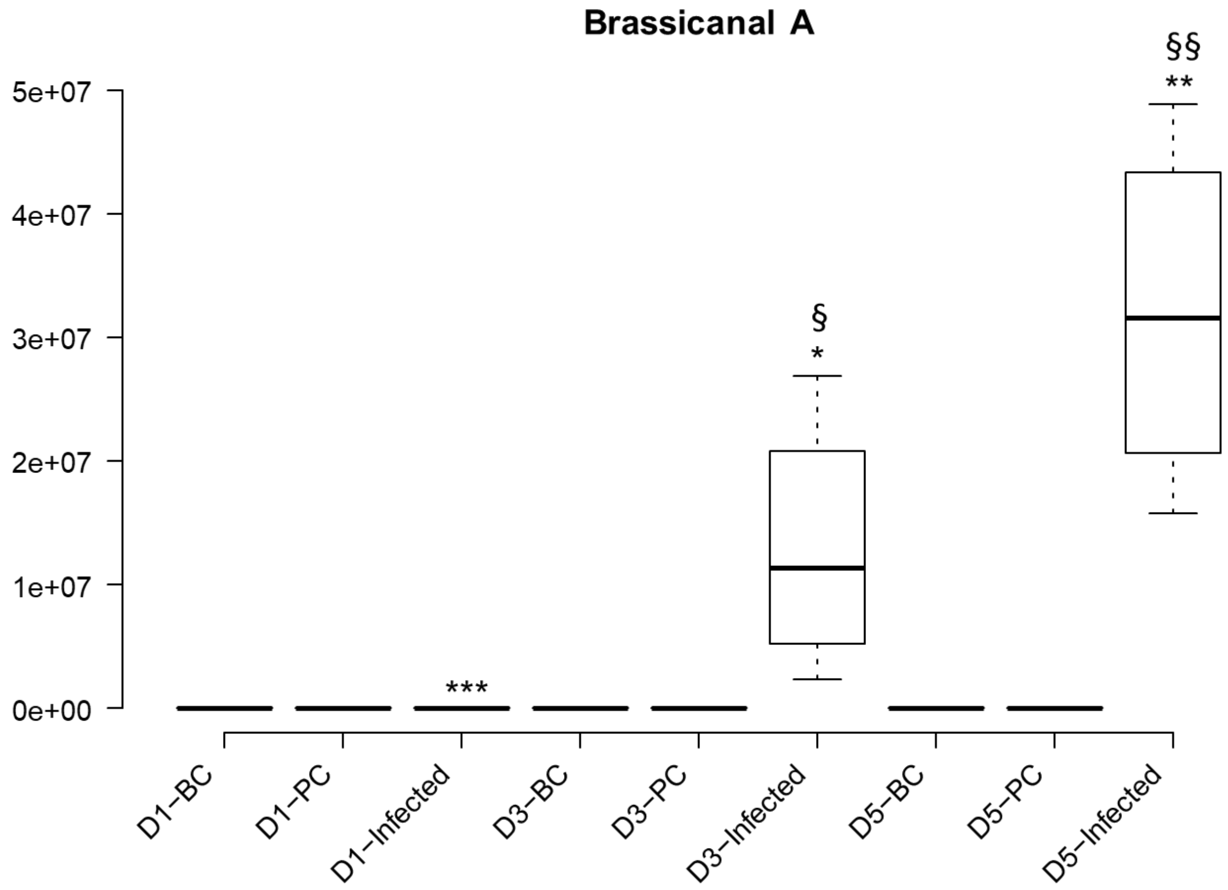

Figure S4-5: Brassilexin (Ion 36)

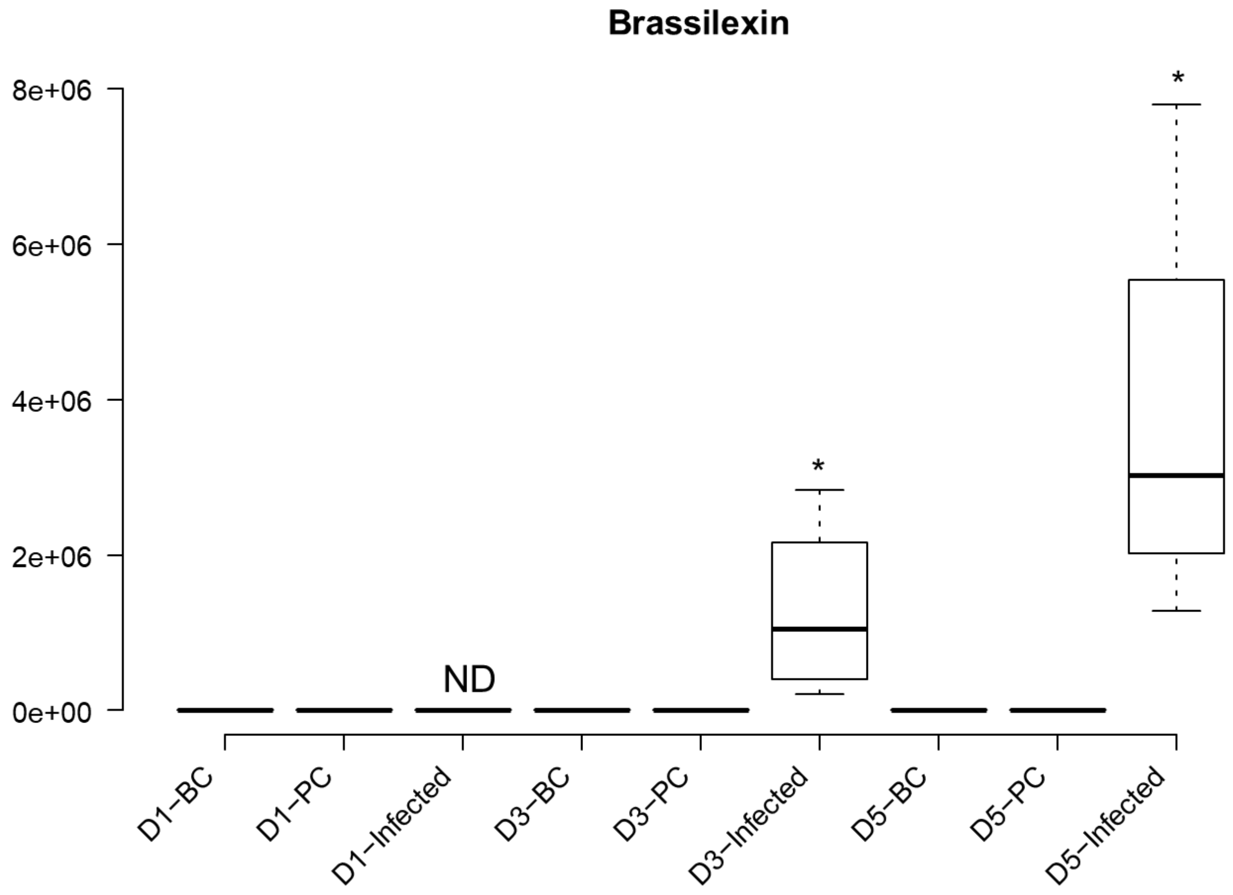

Figure S4-6: Spirobrassinin (Ion 37)

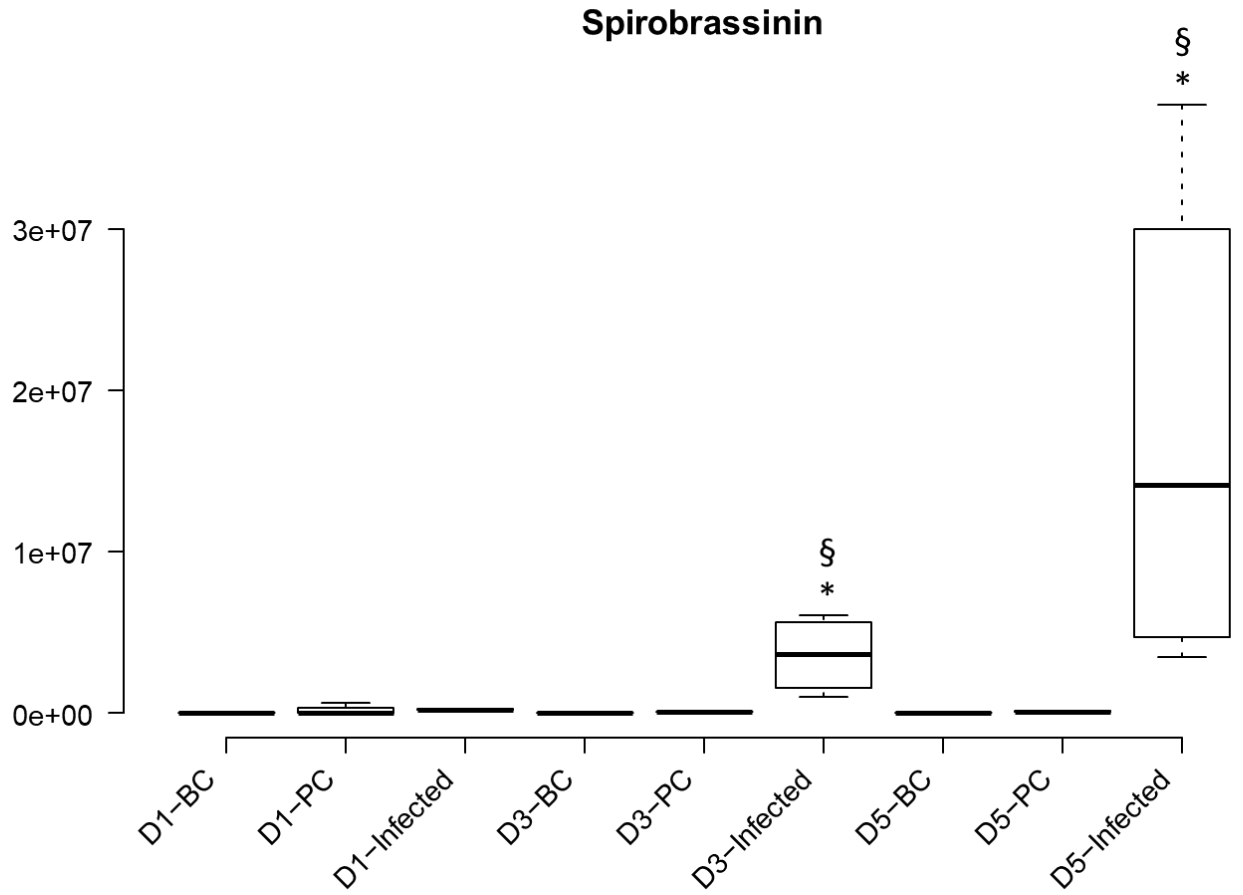

Figure S4-7: 1-Methoxy-3-indolecarboxaldehyde (MICA) (Ion 38)

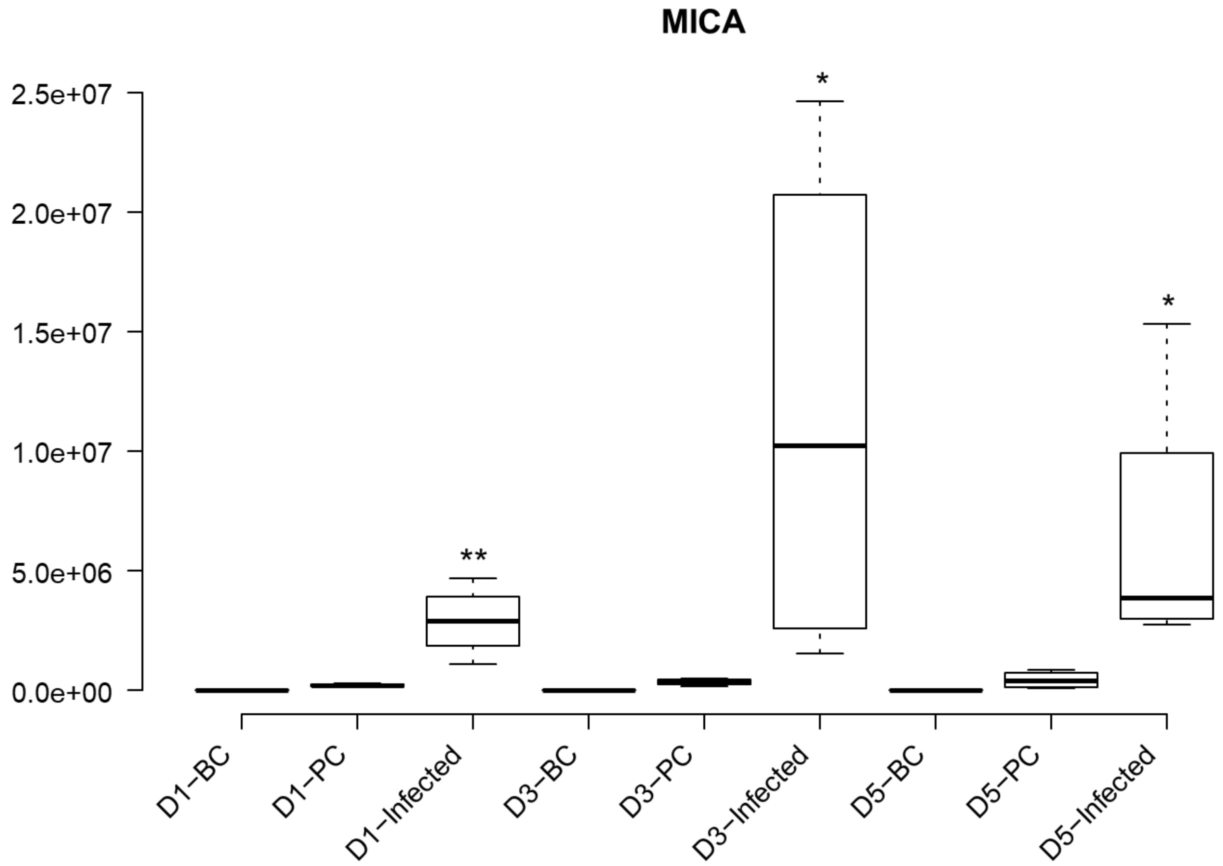

Figure S4-8: 4-Methoxycyclobrassinin (Ion 39)

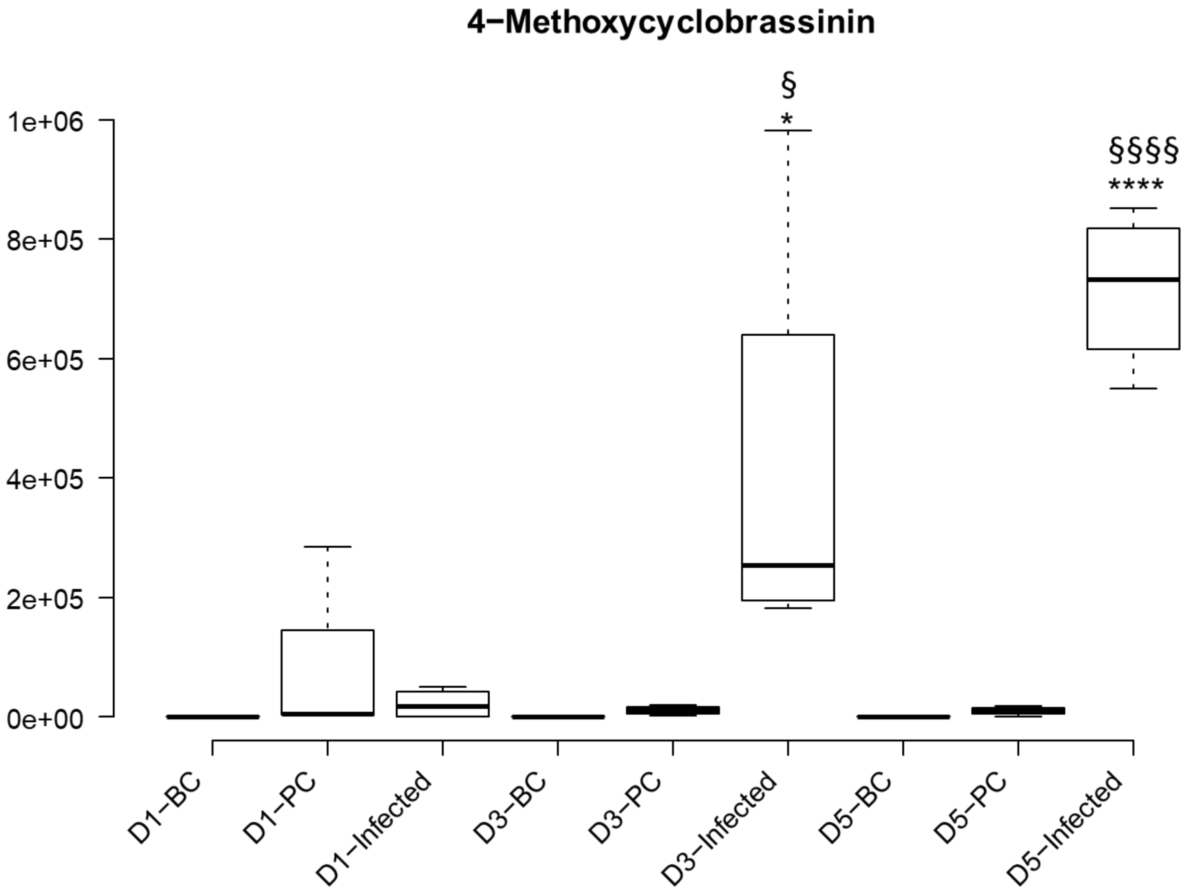

Figure S4-9: Sinalbin B (Ion 40)

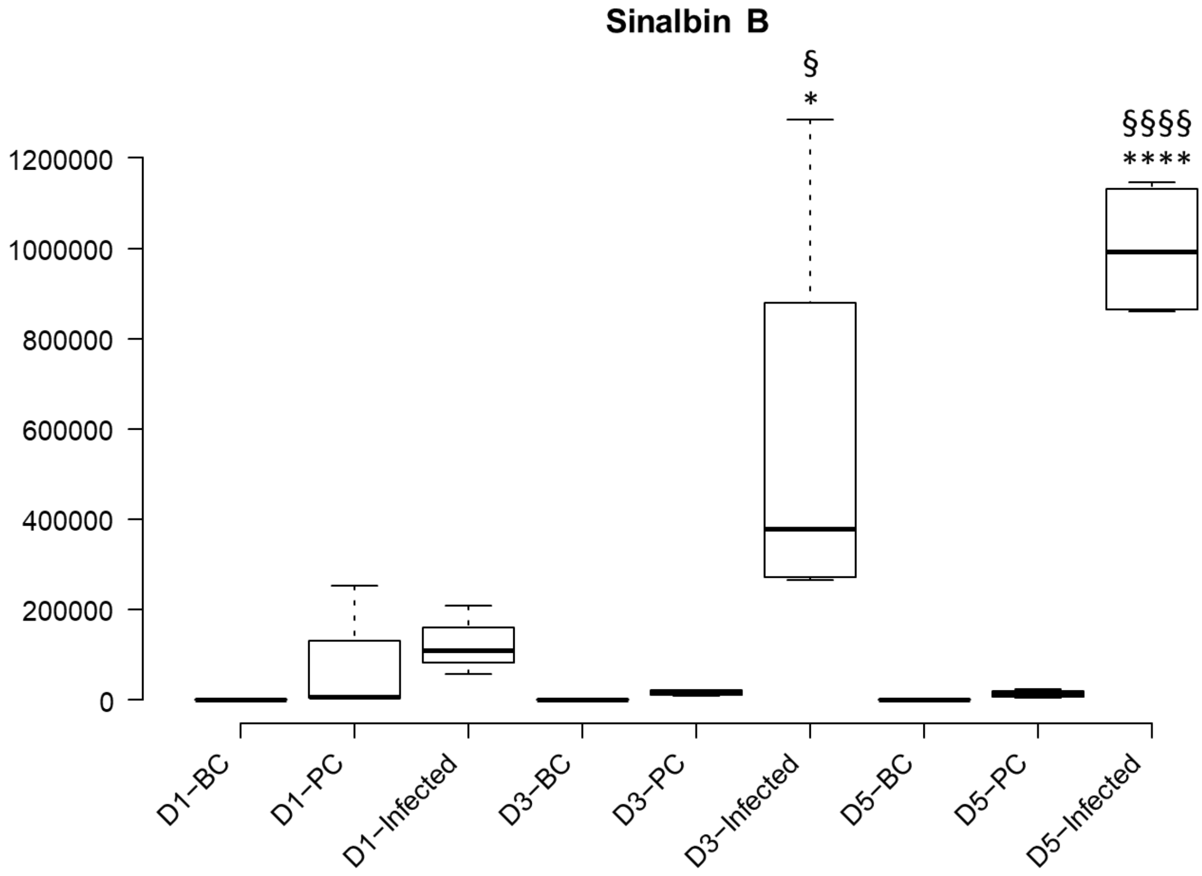

Figure S4-10: Wasalexin A or B (Ion 41)

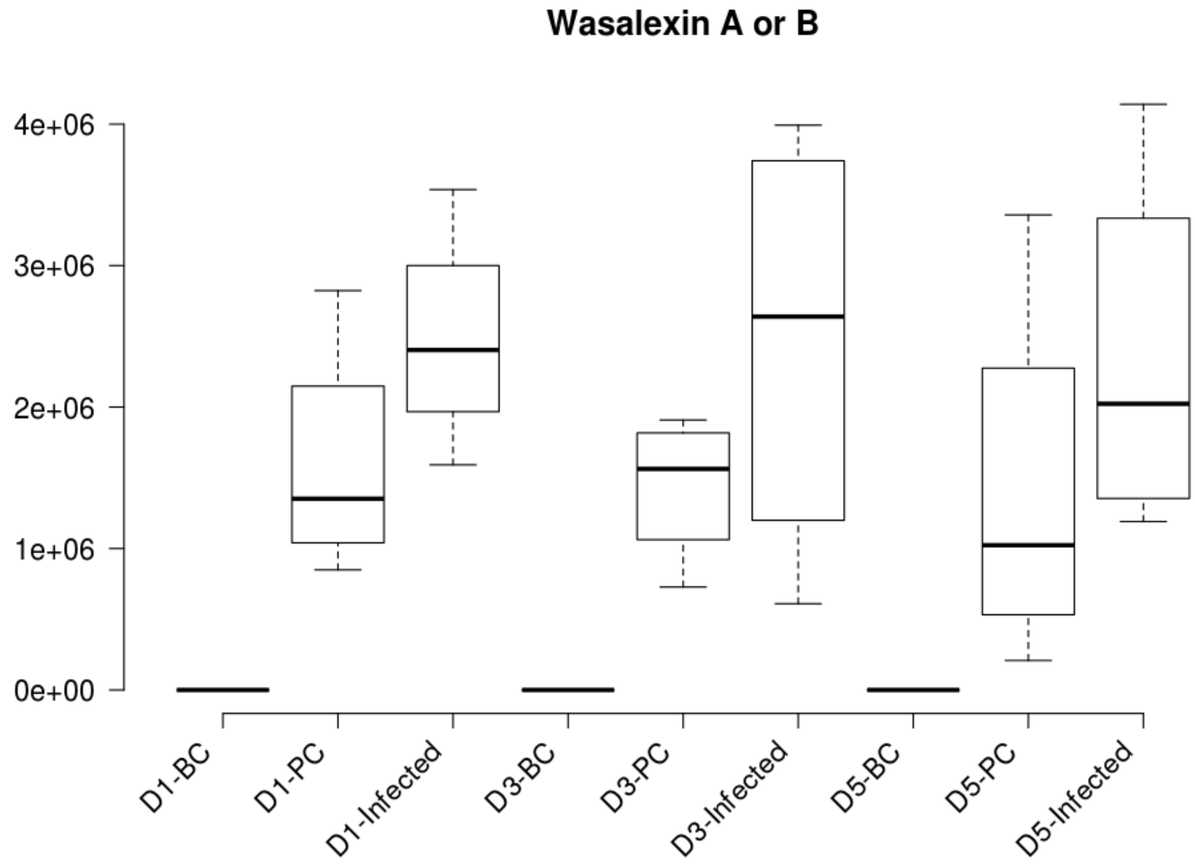

Figure S5: Confirmation (LC-HRMS and MS/MS) of metabolite brassilexin using reference standard. Confirmation of brassilexin with reference standard of brassilexin for retention time (LC-HRMS, A) and MS/MS fragmentation patterns of the precursor ion (B) was carried out by using a D3-Infected as a representative sample.

A

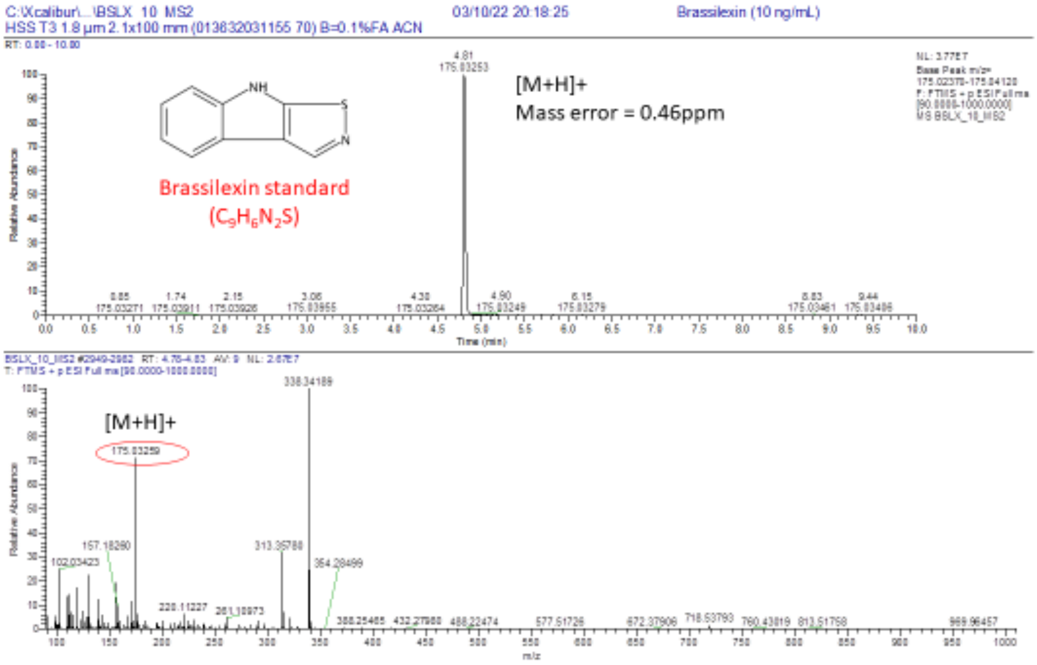

B

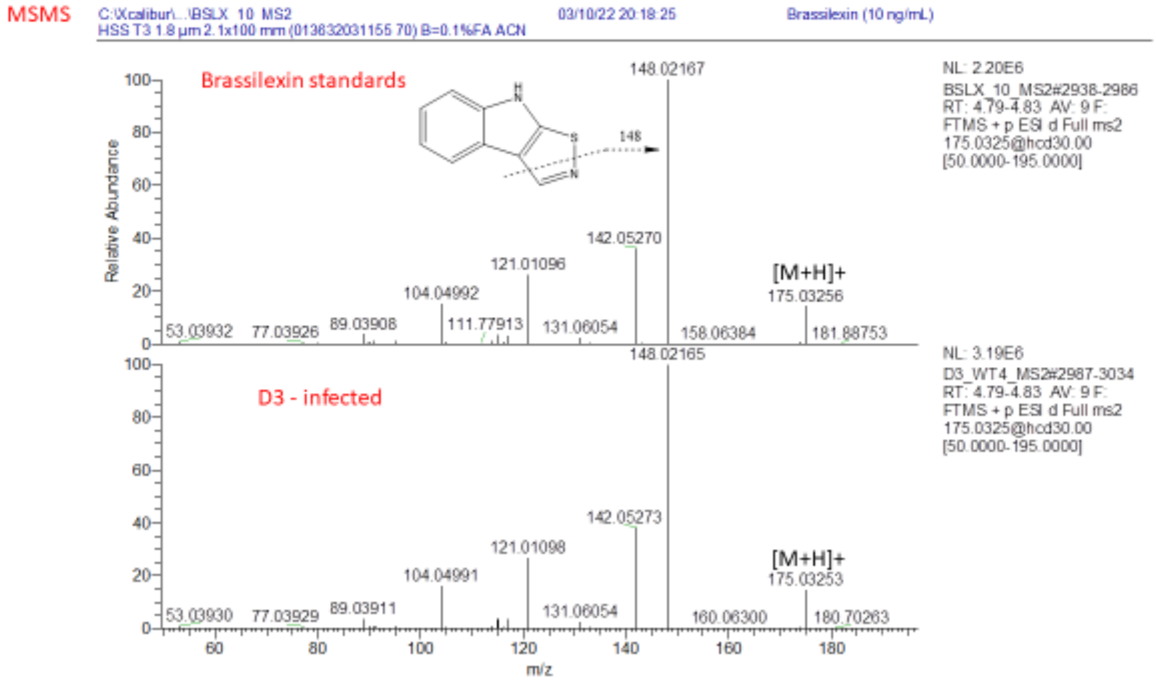
MSMS
C:\Xcalibur\...\BSLX_10_MS2
HSS T3 1.8 µm 2.1x100 mm (013632031155 70) B=0.1%FA ACN
03/10/22 20:18:25
Brassilexin (10 ng/mL)
Brassilexin standards
D3 - infected
148
148.02167
142.05270
121.01096
104.04992
111.77913
131.06054
158.06384
[M+H]+
175.03256
181.88753
53.03932
77.03926
89.03908
NL: 2.20E6
BSLX_10_MS2#2938-2986
RT: 4.79-4.83 AV: 9 F:
FTMS + p ESI d Full ms2
175.0325@hcd30.00
[50.0000-195.0000]
148.02165
142.05273
121.01098
104.04991
131.06054
160.06300
[M+H]+
175.03253
180.70263
53.03930
77.03929
89.03911
NL: 3.19E6
D3_WT4_MS2#2987-3034
RT: 4.79-4.83 AV: 9 F:
FTMS + p ESI d Full ms2
175.0325@hcd30.00
[50.0000-195.0000]
Relative Abundance
m/z
